# Xist exerts gene-specific silencing during XCI maintenance and impacts lineage-specific cell differentiation and proliferation during hematopoiesis

**DOI:** 10.1038/s41467-022-32273-5

**Published:** 2022-08-01

**Authors:** Tianqi Yang, Jianhong Ou, Eda Yildirim

**Affiliations:** 1 Department of Cell Biology, Duke University Medical Center, Durham, NC 27710 USA; 2 Department of Pharmacology and Cancer Biology, Duke University Medical Center, Durham, NC 27710 USA; 3 Duke Regeneration Center, Duke University, Durham, NC 27710 USA; 4 Duke Cancer Institute, Duke University Medical Center, Durham, NC 27710 USA

**Keywords:** Dosage compensation, Long non-coding RNAs, Transcription

## Abstract

X chromosome inactivation (XCI) is a dosage compensation phenomenon that occurs in females. Initiation of XCI depends on Xist RNA, which triggers silencing of one of the two X chromosomes, except for XCI escape genes that continue to be biallelically expressed. In the *soma* XCI is stably maintained with continuous Xist expression. How Xist impacts XCI maintenance remains an open question. Here we conditionally delete Xist in hematopoietic system of mice and report differentiation and cell cycle defects in female hematopoietic stem and progenitor cells (HSPCs). By utilizing female HSPCs and mouse embryonic fibroblasts, we find that X-linked genes show variable tolerance to Xist loss. Specifically, XCI escape genes exhibit preferential transcriptional upregulation, which associates with low H3K27me3 occupancy and high chromatin accessibility that accommodates preexisting binding of transcription factors such as Yin Yang 1 (YY1) at the basal state. We conclude that Xist is necessary for gene-specific silencing during XCI maintenance and impacts lineage-specific cell differentiation and proliferation during hematopoiesis.

## Abstract (web summary)

Here the authors investigate the functional relevance of X-chromosome inactivation (XCI) regulator Xist in hematopoiesis. They find that Xist loss leads to changes in the ratio of hematopoietic progenitor cells and results in chromatin accessibility and transcriptional upregulation on the inactive X chromosome, including XCI escape genes known to be associated with cell cycle and immune response.

## Introduction

X chromosome inactivation (XCI) is a dosage compensation phenomenon that occurs in mammalian females to resolve the inherent X-linked gene dosage imbalance between sexes through transcriptional silencing of one of the two X chromosomes. In mouse, the random form of XCI occurs in the epiblast at embryonic days 4.5–5.5 (E4.5–5.5). This phase is referred as the XCI initiation during which either X chromosome has the equal probability to be chosen as the future inactive X chromosome (Xi). With the establishment of the transcriptionally silent state, the cells enter the XCI maintenance phase in which the same X chromosome is propagated as the Xi in subsequent cell divisions. Proper maintenance of the Xi silent state is essential for female health because XCI maintenance defects have been implicated in various human cancers and developmental diseases^1–6^. Therefore, determining the molecular basis of XCI maintenance is important to provide insights toward development of therapies for XCI-related disorders.

Initiation of XCI depends on Xist RNA which is a ~17 kb transcript that is exclusively expressed from the Xi^7,8^. Xist RNA binds across the Xi in *cis*, and aids recruitment of chromatin regulatory factors such as the Polycomb Repressive complexes (PRC1 and PRC2)^9–11^ that influence the establishment of the silent chromatin state on the Xi. Compared to the active X chromosome (Xa), the Xi exhibits low-level occupancy of active histone marks such as Histone 3 Lysine 4 tri-methylation (H3K4me3) and Histone 3 Lysine 27 acetylation (H3K27ac), and high occupancy of repressive histone marks such as Histone 3 Lysine 27 tri-methylation (H3K27me3)^12^. In addition to the silent chromatin features, the Xi adopts a unique bipartite chromatin organization that presents low chromatin accessibility and lacks topologically associating domains (TADs)^13–15^. Notably, few X-linked genes remain biallelically expressed following the establishment of XCI and are therefore called XCI escape genes. Compared to the genes that are subjective to XCI (which we will refer to as XCI subjective genes), XCI escape genes associate with higher level of active histone modifications and lower level of repressive histone modifications, and these genes often reside at the open chromatin regions^16,17^.

Xist is continuously expressed in the *soma* during the lifetime of the female. Targeted deletion of Xist in various mouse tissues showed partial upregulation of X-linked genes suggesting a role for Xist in XCI maintenance^18–22^. We contributed to these earlier in vivo studies by showing that Xist deletion in hematopoietic stem cells (HSCs) leads to female-specific lethality due to a range of aggressive hematologic malignancies in the form of mixed myelodysplastic syndrome/myeloproliferative neoplasm (MDS/MPN) and chronic myelomonocytic leukemia (CMML)^18^. Unlike the hematopoietic lineage, conditional deletion of Xist in other tissues including skin, gut, brain, and kidney does not lead to early lethality or any obvious illness over the normal mouse life span^19,20^. These findings suggest variable functional roles of Xist in different tissues. However, how X-linked genes are transcriptionally upregulated in Xist-deficient cells and why the hematopoietic system exhibits less tolerance to Xist loss than other tissues remain unclear.

Here we investigate the functional and mechanistic roles of Xist in hematopoietic stem cell and progenitor cells (HSPCs) using the HSC-specific Xist knockout mouse model^18^. We show that Xist deletion leads to lineage-specific differentiation defects and cell cycle alternations in HSPCs. By utilizing mouse HSPCs and clonal mouse embryonic fibroblasts^23,24^ (which are compatible with allele-specific analysis), we further investigate the role of Xist in regulating X-linked gene expression during XCI maintenance. We find that Xist loss leads to preferential upregulation of XCI escape genes compared to XCI subjective genes. Our mechanistic studies further reveal that the high sensitivity of XCI escape genes to transcriptional upregulation in Xist-deficient cells relies on their low H3K27me3 occupancy and high chromatin accessibility that accommodates preexisting binding of transcription factors (TFs), such as Yin Yang 1 (YY1), at the basal state. Collectively, our results indicate that Xist exerts gene-specific regulatory roles in controlling repression of transcription on the Xi during XCI maintenance, which most likely underlie the variability in Xist-deficiency phenotypes between different cell lineages and tissues. Our findings on the role of Xist in regulation of expression of XCI escape and subjective genes will provide insights for treatment strategies of X-linked diseases through manipulating expression of specific X-linked genes on the Xi^25–27^.

## Results

### Xist deletion leads to lineage-specific HSPC differentiation defects

**Fig. 1:**
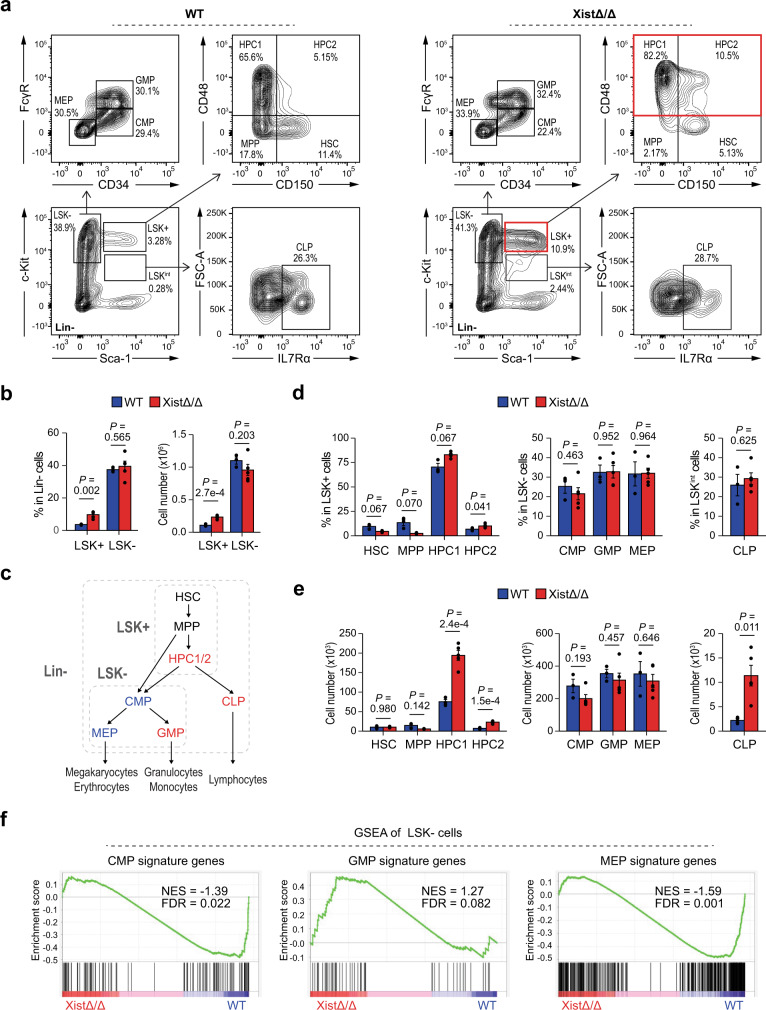
Lineage-specific differentiation defects of hematopoietic progenitors in Xist-deficient mice. **a** Representative FACS analysis of Lin− bone marrow cells from WT and XistΔ/Δ female mice. The HSPC populations with increased percentages in XistΔ/Δ mice compared to WT mice are highlighted in red. **b** FACS analysis of LSK+ and LSK− cells shows increased percentage and number of LSK+ in XistΔ/Δ (*n* = 5) mice compared to WT (*n* = 3) mice. **c** Schematic shows differentiation hierarchy of HSPCs. Cell populations that exhibit increased or decreased differentiation in Xist-deficient female mice are highlighted in red or blue, respectively. **d** FACS analysis of LSK+ and LSK− subpopulations shows increased percentage of HPC1 and HPC2 cells in XistΔ/Δ (*n* = 5) mice compared to WT (*n* = 3) mice. **e** FACS analysis of LSK+ and LSK− subpopulations shows increased number of HPC1, HPC2, and CLP cells in XistΔ/Δ (*n* = 5) mice compared to WT (*n* = 3) mice. **f** Gene set enrichment analysis (GSEA) shows decreased expression of CMP and MEP signature genes and increased expression of GMP signature genes in XistΔ/Δ LSK− cells in comparison to WT LSK− cells. Normalized enrichment scores (NES) and false discovery rates (FDR) are as indicated and FDR < 0.05 is defined as significant. All error bars represent standard error of the mean (SEM), and data are presented as mean values +/− SEM (**b**, **d**, **e**). *P*-values were calculated using two-tailed unpaired Student’s *t*-test. Source data are provided as a Source data file.

Deletion of Xist in HSCs leads to progressive hematopoietic defects resulting in hematologic malignancies and lethality in female mice^18^. In contrast, deletion of Xist in differentiated hematopoietic cells such as the pro-B lymphocytes does not result in any obvious disease phenotype or lethality^28^. This suggests that the role of Xist during hematopoiesis is associated with differentiation status of cells. To investigate the functional role of Xist in HSPCs, we utilized female mice at early disease stage (3-month-old) and quantified the number and percentage of different HSPC populations within lineage-depleted (Lin−) bone marrow cells by fluorescence-activated cell sorting (FACS) analyses^29^ (Supplementary Fig. 1a). Consistent with our previous findings^18^, we observed a significant increase in the percentage (~2.7 fold; *P* = 0.002) and number (~2.2 fold; *P* = 2.7e–4) of LSK+ (Lin^−^c-Kit^+^Sca-1^+^) cells in Xist homozygous knockout (XistΔ/Δ) mice compared to their wild-type (WT) littermates (Fig. 1a, b). LSK+ cells are composed of four subpopulations that can be characterized using SLAM cell-surface markers^30^. These subgroups include HSCs (Lin^−^c-Kit^+^Sca-1^+^CD150^+^CD48^-^), multipotent progenitor cells (MPPs) (Lin^−^c-Kit^+^Sca-1^+^CD150^-^CD48^-^) and two hematopoietic progenitor populations with restricted pluripotency, HPC1 (Lin^−^c-Kit^+^Sca-1^+^CD150^−^CD48^+^) and HPC2 (Lin^−^c-Kit^+^Sca-1^+^CD150^+^ CD48^+^) (Fig. 1a, c). We detected significant increase in the number of HPC1 (~2.6 fold; *P* = 2.4e–4) and HPC2 (~3.2 fold; *P* = 1.5e–4) cells in XistΔ/Δ female mice (Fig. 1e).

To investigate whether expansion of HPC1/2 cells affected downstream cell differentiation, we quantified lineage-specific hematopoietic progenitor populations by FACS^30^ (Fig. 1a, c). We found that Xist depletion resulted in a significant increase (~5.2 fold; *P* = 0.011) in the number of common lymphoid progenitors (CLP; Lin^−^ c-Kit^+^ Sca-1^int^IL7Rα^+^) (Fig. 1e). In contrast, the percentage and the number of LSK− (Lin^−^c-Kit^+^Sca-1^−^) myeloid progenitors, including common myeloid progenitors (CMP; Lin^−^c-Kit^+^Sca-1^−^CD34^+^FcγR^int^), granulocyte-monocyte progenitors (GMP; Lin^−^c-Kit^+^Sca-1^−^CD34^+^FcγR^+^) and megakaryocyte–erythroid progenitors (MEP; Lin^−^c-Kit^+^Sca-1^−^CD34^−^FcγR^−^), were comparable in WT and XistΔ/Δ mice (Fig. 1d, e). Despite the lack of changes in the number of myeloid progenitor cell populations, Gene Set Enrichment Analyses (GSEA)^31^ of their signature genes (Supplementary Fig. 2) using LSK− cell RNA-seq data showed that Xist deletion resulted in a slight upregulation in GMP signature gene expression (NES = 1.27, FDR = 0.082) and significant reduction in the expression of CMP (NES = −1.39, FDR = 0.022) and MEP (NES = −1.59, FDR = 0.001) signature genes (Fig. 1f). Based on these results, we concluded that Xist deletion facilitates lymphoid progenitor differentiation and hinders myeloid progenitor differentiation towards the megakaryocyte and erythrocyte lineage (Fig. 1c).

### Xist deletion leads to increased cycling of hematopoietic progenitor cells

**Fig. 2:**
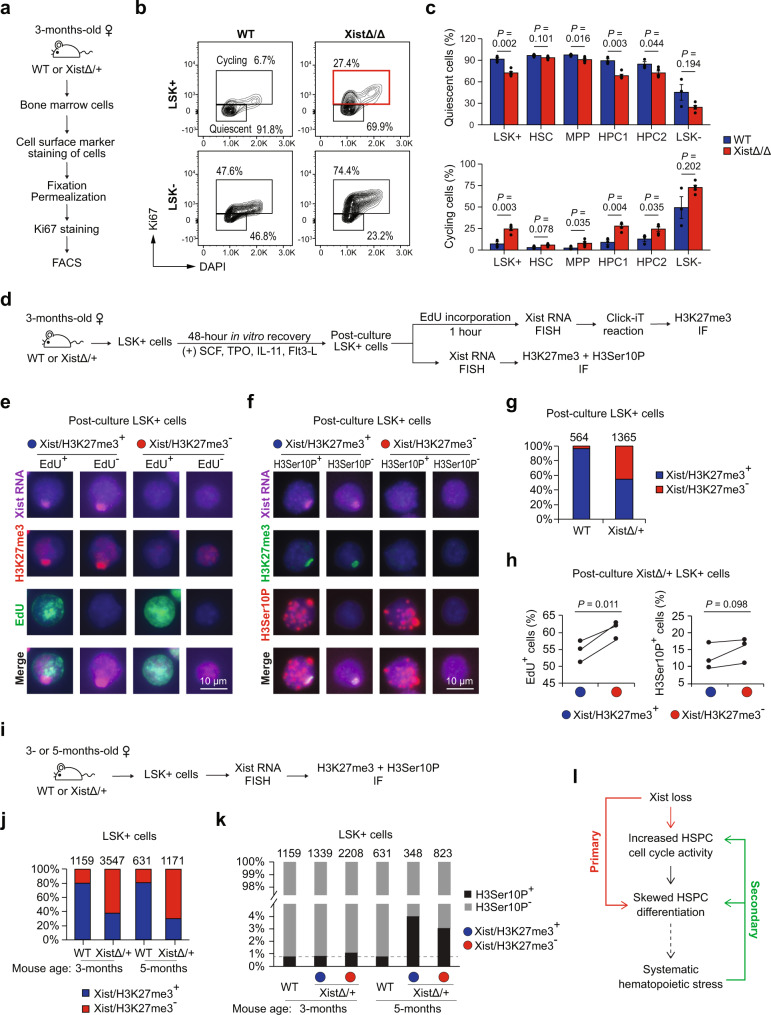
Increased cycling of hematopoietic progenitor cells in Xist-deficient mice. **a** Schematic shows experimental design for Ki67 cell cycle assay. **b** Representative FACS analysis of quiescent and cycling cells in WT and XistΔ/Δ LSK+ and LSK− cells based on Ki67 staining. **c** FACS analysis shows increase in the percentage of cycling and decrease in the percentage of quiescent LSK+, MPP, HPC1, and HPC2 cells in XistΔ/Δ mice (*n* = 5) in comparison to WT (*n* = 3) female mice. Data are presented as mean values +/− SEM. **d** Schematic shows experimental design to study replication and mitosis of post-culture LSK+ cells utilizing Click-iT EdU assay and H3Ser10P IF staining. **e** Representative images of sequential Xist RNA FISH, Click-iT EdU reaction, and H3K27me3 IF staining in post-culture LSK+ cells after EdU incorporation. **f** Representative images of sequential Xist RNA FISH followed by H3K27me3 and H3Ser10P IF staining in post-culture LSK+ cells. **g** Analysis of the percentage of Xist/H3K27me3^+^ and Xist/H3K27me3^−^ cells in post-culture WT and XistΔ/+ LSK+ cells. **h** Analysis of the percentage of EdU^+^ and H3Ser10P^+^ cells in post-culture Xist/H3K27me3^+^ and Xist/H3K27me3^−^ XistΔ/+ LSK+ cells. Data points for cells obtained from the same mouse are connected by a line. At least 200 cells were analyzed for each mouse in each assay. **i** Schematic shows experimental design of H3Ser10P IF staining-based mitosis assay using LSK+ cells directly isolated from the mouse bone marrow. See Supplementary Fig. 3 for representative staining images. **j** Analysis of the percentage of Xist/H3K27me3^+^ and Xist/H3K27me3^−^ cells in LSK+ cells directly isolated from 3- and 5-month-old WT and XistΔ/+ mice. **k** Analysis of the percentage of H3Ser10P^+^ or H3Ser10P^-^ cells in LSK+ cells isolated from 3- or 5-month-old WT and XistΔ/+ mice. **l** Working model shows how hematopoietic malignancies develop and progress in Xist-deficient female mice. Primary effects of Xist deletion and secondary effects of systematic hematopoietic stress are highlighted in red and green, respectively. Analyzed cell numbers are indicated above each bar graph (**g**, **j**, **k**). *P*-values were calculated using two-tailed unpaired (**c**) or paired (**h**) Student’s *t*-test. Source data are provided as a Source data file.

Differentiation and proliferation of HSPCs are finely controlled by cell cycle checkpoints^32^. Given the alternations in proliferation and differentiation of Xist-deficient HSPCs (Fig. 1), we investigated how Xist loss impacts cell cycle by staining bone marrow cells for the cell proliferation marker, Ki67, followed by FACS analyses of quiescent cells at G0 phase (low Ki67) and cycling cells at G1/S/G2/M phase (high Ki67) (Fig. 2a, b and Supplementary Fig. 1b). We found that Xist deletion resulted in significant increase in the cell cycle frequency of LSK+ subpopulations including MPP (~3.4 fold; *P* = 0.034), HPC1 (~3.1 fold; *P* = 0.004), and HPC2 (~1.9 fold; *P* = 0.035) cells (Fig. 2c). In contrast, the cell cycle frequency of Xist-deficient LSK− cells did not significantly change (Fig. 2c).

Cytological analyses of the spleen and bone marrow cells in our earlier study have revealed increased mitotic figures in HSC-specific Xist-deficient mice^18^. In addition, it has been reported that Xist-deficient fibroblasts exhibit delayed replication of Xi and extended S phase^33^. These data prompted us to further determine how Xist affects replication and mitosis of LSK + HSPCs by performing Click-iT EdU assay^34^ and immunofluorescence (IF) staining against a mitotic marker, Histone 3 Serine 10 phosphorylation (H3Ser10P) (Fig. 2d–f). LSK+ cells used in these assays, which we referred to as post-culture LSK+ cells, were first sorted from the mouse bone marrow and then recovered in culture for 48 h in the presence of four stem cell cytokines (SCF, TPO, IL-11, Flt3-L)^35^ (Fig. 2d). By sequential Xist RNA Fluorescence In Situ Hybridization (FISH) and H3K27me3 IF staining, we showed that more than 98% of WT post-culture LSK+ cells were positive for Xist cloud that overlapped with H3K27me3 foci (Xist/H3K27me3^+^) (Fig. 2g). In contrast, about half of the post-culture LSK+ cells from Xist heterozygous knockout (XistΔ/+) mice lacked Xist cloud and H3K27me3 foci (Xist/H3K27me3^−^) (Fig. 2g). Among the post-culture XistΔ/+ LSK+ cells, we found the Xist/H3K27me3^−^ cells exhibited significantly higher frequency of EdU incorporation (EdU^+^) (*P* = 0.011) and slightly higher percentage of H3Ser10P stained (H3Ser10P^+^) cells (*P* = 0.098) than the Xist/H3K27me3^+^ cells (Fig. 2h). It is expected that Xist/H3K27me3^−^ XistΔ/+ cells carry Xist deletion on the Xi (Xi^ΔXist^), while Xist/H3K27me3^+^ XistΔ/+ cells carry Xist deletion on the Xa (Xa^ΔXist^) and act as internal controls for Xi^ΔXist^ LSK+ cells obtained from the same mouse. Thus, we concluded that Xist deletion leads to increase in the frequency of HSPCs at S and M phase.

Interestingly, when we performed sequential Xist RNA-FISH and H3K27me3 IF staining in LSK+ cells that were directly isolated from the mouse bone marrow (Fig. 2i and Supplementary Fig. 3), we detected ~20% of WT LSK+ cells to be Xist/H3K27me3^−^ (Fig. 2j). This data is in line with the findings of an earlier study^36^ suggesting that there is likely a cross talk between XCI status and cellular stress in vivo, which we referred to as systematic hematopoietic stress. Notably, compared to WT cells, the percentage of H3Ser10P^+^ cells was drastically higher in XistΔ/+ cells independent of Xist expression or presence of H3K27me3 foci at a later disease stage (5-month-old) (Fig. 2k). These results indicated that phenotypes of Xist-deficient HSPCs, including skewed differentiation ratios and increased cycling frequency, are not only a primary effect of Xist deletion but also a secondary effect in response to systematic hematopoietic stress that accumulates over time (Fig. 2l).

### Xist deletion results in partial transcriptional upregulation of X-linked genes in HSPCs

**Fig. 3:**
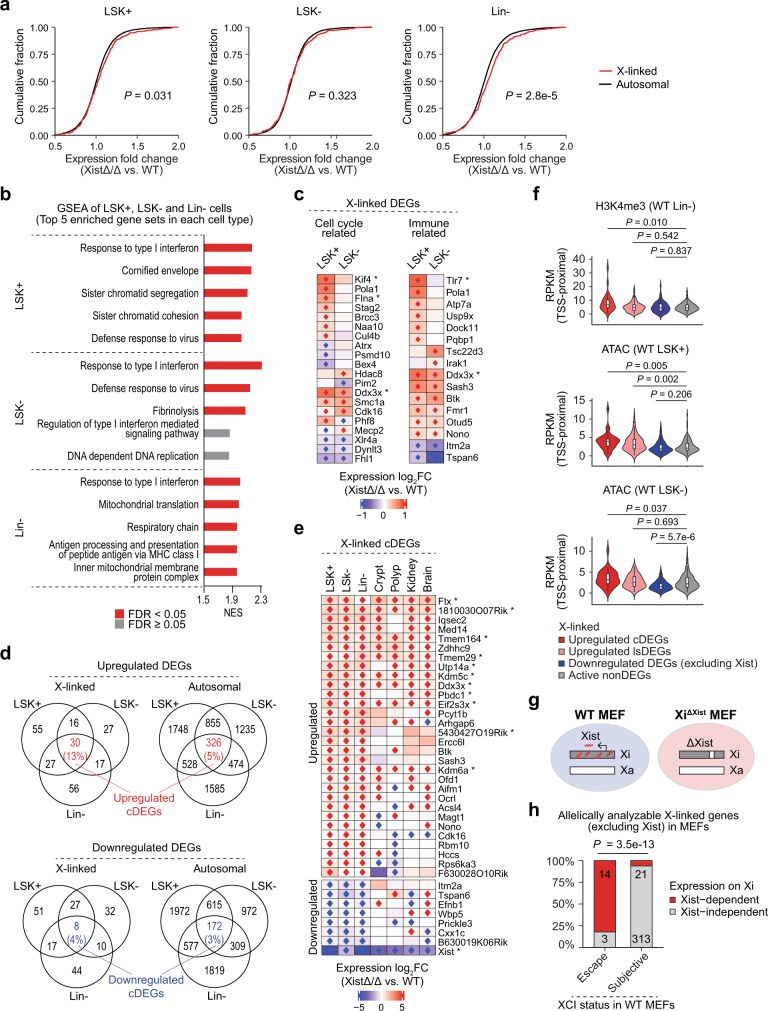
Partial transcriptional upregulation of X-linked genes in Xist-deficient cells. **a** Cumulative distribution fraction (CDF) plot of expressional fold change of X-linked and autosomal genes between XistΔ/Δ and WT mice show increase in overall expression level of X-linked genes in XistΔ/Δ LSK+ and Lin− cells. Genes with FPKM ≥ 1 were used for analysis. **b** GSEA of LSK+, LSK−, and Lin− differential expression profiles. Top 5 gene sets enriched in XistΔ/Δ cells (ranked by normalized expression score (NES)) were plotted. For complete GSEA result analysis see Supplementary Data 1. **c** Heat map shows expression log2 fold change (log_2_FC) of cell cycle- and immune-related X-linked DEGs in LSK+ and LSK− cells. Genes that are transcriptionally upregulated (red) or downregulated (blue) in both biological replicates (DEGs) are marked by rhombuses. Genes that have been reported to escape XCI in mouse cells are marked with asterisk (*)^51,53^. **d** Venn diagrams show overlap between X-linked or autosomal DEGs in LSK+, LSK−, and Lin− cells. Upregulated or downregulated DEGs that are shared in all three cell types (cDEGs) are highlighted in red and blue, respectively. **e** Heat map shows expression log_2_FC of X-linked cDEGs in bone marrow cells (LSK+, LSK−, and Lin− cells) in comparison to cells from other tissues including brain, gut (crypt and polyp), and kidney^20,28^. DEGs and XCI escape genes are marked as in (**c**). **f** Violin and box plots compare H3K4me3 or ATAC signal density within TSS-proximal regions of different types of X-linked DEGs in comparison to active nonDEGs in WT LSK+, LSK−, or Lin− cells. See Supplementary Data 2 and 3 for lists of genes and peaks within each group. RPKM, Reads Per Kilobase per Million mapped reads. **g** Schematic shows genotype of clonal WT and Xi^ΔXist^ MEF cell lines. **h** Analysis of the correlation between Xist-dependent genes and XCI escape genes in MEFs. Signals obtained from two biological replicates were averaged for making the plots (**f**). Boxplots are described in “Methods” (**f**). *P*-values were calculated using two-tailed Kolmogorov–Smirnov statistic (**a**), two-tailed unpaired Student’s *t*-test (**f**) or two-tailed Fisher’s exact test (**h**).

Previous microarray-based transcriptional profiling of multiple hematopoietic cell populations have shown that transcription of several X-linked genes was progressively upregulated during disease progression in Xist-deficient female mice^18^. Here, we utilized RNA-seq and generated a comprehensive transcription profile for Lin−, LSK+, and LSK− cells in WT and Xist-deficient mice (*n* = 2 biological replicates per genotype). Cumulative distribution fraction (CDF) analysis of expression fold changes of all transcriptionally active genes (FPKM ≥ 1) revealed a significant right shift of X-linked CDF curve compared to the autosomal CDF curve in LSK+ cells (*P* = 0.031) (Fig. 3a). This finding demonstrated that Xist deletion leads to dosage compensation defects in LSK+ cells resulting in an overall increase in expression on the X chromosome. In addition, the right shift of X-linked CDF curve became more pronounced in Lin− cells (*P* = 2.8e−5) (Fig. 3a), suggesting accumulation of dosage compensation defects as the cells differentiate. In contrast, the X-linked CDF curve was comparable to the autosomal CDF curve in LSK− cells (Fig. 3a), supporting our findings that myeloid and lymphoid progenitors exhibit different tolerance to Xist loss (Fig. 1).

To determine the biological function of transcriptionally altered genes in Xist-deficient HSPCs, we performed GSEA of differential expression profiles of HSPC subpopulations using ontology gene sets. The result revealed that expression of genes that associate with Type-I interferon-mediated immune response was significantly enriched (FDR < 0.05) in all three Xist-deficient HSPC populations (Fig. 3b and Supplementary Data 1). On the other hand, expression of genes related to chromatin segregation, DNA replication, and cell division was significantly enriched (FDR < 0.05) in LSK+ cells but not in LSK− cells in Xist-deficient mice (Fig. 3b and Supplementary Data 1). To narrow down candidate genes that might be causally linked to the phenotype of Xist-deficient female mice, we determined differentially expressed genes (DEGs) based on their upregulated or downregulated expression between WT and XistΔ/Δ cells in both biological replicates (Supplementary Data 1). We next focused on X-linked DEGs with cell cycle- and immune-related functions in gene ontology (GO) analysis (Supplementary Data 1). We found that genes in both categories presented more transcriptional upregulation in LSK+ cells compared to LSK− cells in Xist-deficient mice (Fig. 3c). These results suggested that the number of transcriptionally upregulated X-linked genes with immune- and cell cycle-related functions might influence the severity of the functional defects in Xist-deficient HSPCs. This finding prompted us to further investigate the type of genes that are transcriptionally upregulated upon Xist loss and the mechanisms behind the process.

### XCI escape genes are highly susceptible to transcriptional upregulation upon Xist loss

We next compared DEGs in different HSPC populations. We grouped DEGs that are shared by all three HSPC populations as common DEGs (cDEGs), while the rest as lineage-specific DEGs (lsDEGs) (Fig. 3d and Supplementary Data 1). We found that ~13% of transcriptionally upregulated X-linked genes to be cDEGs (Fig. 3d). Interestingly, nearly one third of the upregulated X-linked cDEGs (11/30) (*Ddx3x, Eif2s3x, Kdm6a/Utx, Kdm5c/Jarid1c, Ftx, Tmem164, Tmem29, 1810030O07Rik, Utp14a, Pbdc1,* and *5430427O19Rik*) were previously characterized as XCI escape genes in mouse brain, spleen, or ovary^37^ (Fig. 3e). Furthermore, these XCI escape genes showed increased expression upon Xist loss in tissues that have been characterized to exhibit high tolerance to Xist deficiency, such as the gut (crypt and polyp), kidney, and brain^20,28^ (Fig. 3e). In contrast to the upregulated X-linked DEGs, no more than 5% of the downregulated X-linked DEGs or up/downregulated autosomal DEGs were shared by the three HSPC populations (Fig. 3d), and there was no consistency in the expressional changes of these genes between different tissues (Supplementary Fig. 4). These data suggested that transcriptional upregulation of a subset of X-linked genes, especially the ones that have been characterized as XCI escape genes^37^, is a direct effect of Xist loss rather than a secondary response to systematic hematopoietic stress.

Due to the lack of single nucleotide polymorphisms (SNPs) between 129Sv4/Jae and C57BL/6J mouse stains and the mosaicism of cells with either paternal or maternal X chromosome being silenced in the bone marrow cells, we could not examine the XCI status of each X-linked gene based on our HSPC RNA-seq data. To overcome this limitation, we examined WT HSPCs for epigenetic features known to be associated with XCI escape genes^16^. We found upregulated X-linked cDEGs presented significantly higher H3K4me3 occupancy and chromatin accessibility within ±3 kb from their transcription start sites (TSS-proximal region) compared to the background, which was represented by the signal detected surrounding the transcriptionally active but unchanged X-linked genes (active nonDEGs) (Fig. 3f and Supplementary Data 2 and 3). In contrast, X-linked upregulated lsDEGs and downregulated DEGs (excluding Xist) presented similar H3K4me3 level and chromatin accessibility as the background in LSK− and Lin− cells (Fig. 3f and Supplementary Data 2 and 3). These results indicated that upregulated X-linked cDEGs are enriched in genes that reside at the active chromatin regions similar to XCI escape genes.

To further provide direct evidence supporting that XCI escape genes are highly susceptible to transcriptional upregulation upon Xist loss, we performed RNA-seq using clonal WT and Xi^ΔXist^ female mouse embryonic fibroblast (MEF) cell lines^22,24^. In these cells, X chromosomes are inherited from the *Mus musculus* (129Sv4/Jae, Xi, or Xi^ΔXist^) and the *Mus castaneous* (CAST/EiJ, Xa) mouse strains, respectively (Fig. 3g). Based on SNP differences, we quantified RNA-seq reads aligned to Xi (Reads_Xi_) or Xa (Reads_Xa_) for each X-linked gene (Supplementary Data 4). We defined genes with Reads_Xi_ no less than 5% of Reads_Xa_ (Reads_Xi_/Reads_Xa_ ≥ 0.05) as XCI escape genes and the others as XCI subjective genes. We next compared expression d-score (Reads_Xi_/(Reads_Xi_ + Reads_Xa_) – 0.5) for each gene in WT and Xi^ΔXist^ MEFs. X-linked genes with increased expression d-score (Xi^ΔXist^ d-score – WT d-score ≥ 0.03) are expected to show upregulated Xi-specific expression upon Xist depletion and therefore were referred to as Xist-dependent genes, while the remaining were referred to as Xist-independent genes (Supplementary Data 4). Among the 352 X-linked genes that were allelically analyzable in both WT and Xi^ΔXist^ MEFs, we detected 18 XCI escape genes (including Xist) and 334 XCI subjective genes (Supplementary Data 4). We found 14 of the 18 XCI escape genes were Xist-dependent, while only 21 of the 334 XCI subjective genes were Xist-dependent (Supplementary Fig. 5 and Supplementary Data 4). By Fisher’s exact test, we showed that the association between XCI escape genes and Xist-dependent genes was statistically significant (*P* = 3.5e−13) (Fig. 3h).

### Loss of H3K27me3 does not exclusively correlate with transcriptional upregulation of X-linked genes in Xist-deficient HSPCs

**Fig. 4:**
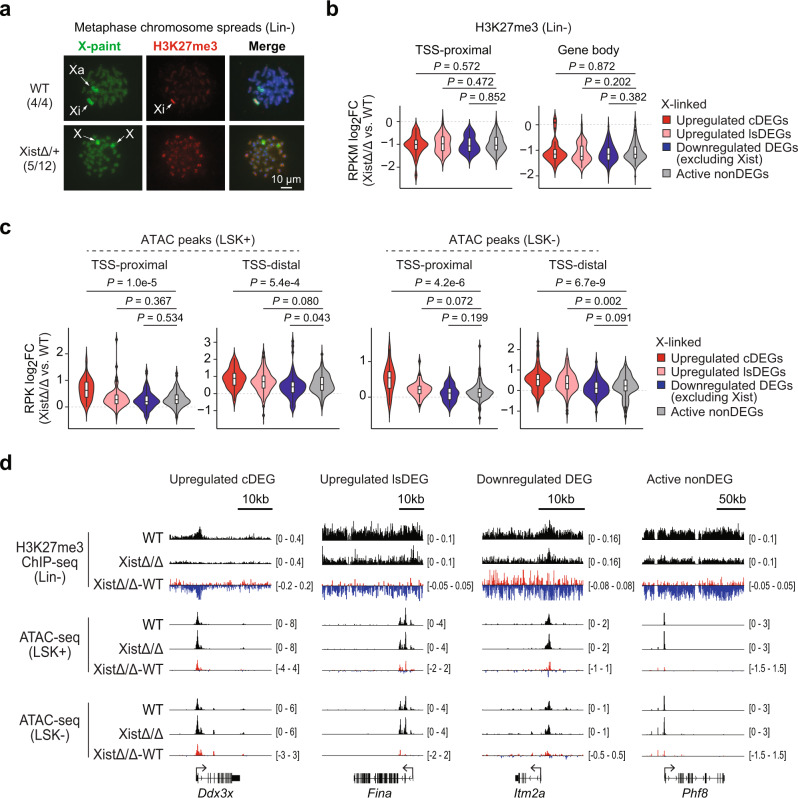
Transcriptional upregulation of X-linked genes is associated with increase in chromatin accessibility in Xist-deficient cells. **a** Representative images of sequential H3K27me3 IF staining and X-paint DNA FISH of metaphase chromosome spreads of Lin− cells isolated from WT and XistΔ/+ female mice. The fractions of metaphase chromosome spreads with similar staining results are as indicated. **b** Violin and box plots show changes in H3K27me3 density within TSS-proximal and gene body regions of different types of DEGs as well as active nonDEGs on the X chromosome between XistΔ/Δ and WT Lin− cells. RPKM, Reads Per Kilobase per Million mapped reads. **c** Violin and box plots show changes in ATAC peak intensity within TSS-proximal and TSS-distal regions of different types of DEGs as well as active nonDEGs on the X chromosome between XistΔ/Δ and WT LSK +/− cells. RPK, Reads Per Kilobase. **d** Representative genome tracks show distribution of H3K27me3 ChIP-seq (in Lin− cells) and ATAC-seq (in LSK+ and LSK− cells) signal across typical X-linked upregulated cDEG (*Ddx3x*), upregulated lsDEG (*Fina*), downregulated DEG (*Itm2a*) and active nonDEG (*Phf8*) in WT and XistΔ/Δ cells. See Supplementary Data 2 (**b**) and 3 (**c**) for a list of genes and/or peaks belonging to each group. Signals obtained from two biological replicates were averaged for making the plots (**b**, **c**). Boxplots are described in “Methods”. – values were calculated using two-tailed unpaired Student’s *t*-test.

Recruitment of the PRC2 is one of the early XCI initiation events that triggers heterochromatinization of the Xi through tri-methylation of H3K27 (H3K27me3)^11,12,38^. Earlier studies have shown that Xist deletion during XCI maintenance leads to loss of H3K27me3 on the Xi^20,21,24,33,39^. Here, we also observed loss of Xist cloud together with H3K27me3 foci in nearly half of the XistΔ/+ LSK+ cells (Fig. 2g, j). We further validated this finding by performing sequential H3K27me3 IF staining and X-paint DNA FISH on metaphase chromosome spreads of XistΔ/+ Lin− cells and detected loss of H3K27me3 on one of the two X chromosomes (Fig. 4a). To investigate whether reduced H3K27me3 occupancy is associated with upregulated expression of X-linked genes in Xist-deficient cells, we performed H3K27me3 ChIP-seq in Lin− cells. We detected a drastic reduction in H3K27me3 occupancy across X-linked genes in XistΔ/Δ Lin− cells compared to WT cells (Supplementary Fig. 6). However, H3K27me3 loss at TSS-proximal and gene body regions was observed for not only upregulated DEGs (e.g., *Ddx3x* and *Fina*) but also downregulated DEGs (e.g., *Itm2a*) and active nonDEGs (e.g., *Phf8*) on the X chromosome (Fig. 4b, Supplementary Fig. 6b and Supplementary Data 2). This data suggested that H3K27me3 loss is not exclusively associated with transcriptional upregulation of X-linked genes in Xist-deficient cells.

### Transcriptionally upregulation of X-linked genes is associated with increased chromatin accessibility in Xist-deficient HSPCs

An earlier study has shown that Xist-deficient MEFs exhibit increased chromatin accessibility at selective sites on the X chromosome^40^. To determine how Xist deletion affects chromatin accessibility on the X chromosome in hematopoietic cells and whether this process is associated with transcriptional upregulation of X-linked genes, we performed ATAC-seq^41^ in WT and XistΔ/Δ LSK+ and LSK− cells. The results showed that Xist deletion did not lead to drastic change in the overall chromatin accessibility (Supplementary Fig. 7a). Instead, it resulted in slight increase in the number and intensity of ATAC peaks on both X chromosome and autosomes (Supplementary Fig. 7b, c and Supplementary Data 3). Importantly, we found that the increase in intensity of ATAC peaks annotated to the upregulated X-linked cDEGs (e.g., *Ddx3x*) was significantly larger than that of active X-linked nonDEGs at both TSS-proximal and TSS-distal (>3 kb from TSS) regions (*P* < 0.001) (Fig. 4c, d and Supplementary Data 3). A milder increase in chromatin accessibility was also observed for upregulated X-linked lsDEGs especially at the TSS-distal regions (e.g., *Fina*) (Fig. 4c, d and Supplementary Data 3). Therefore, we concluded that Xist deletion results in preferential increase in chromatin accessibility at sites that associate with transcriptionally upregulated X-linked genes.

### Basal H3K27me3 level and chromatin accessibility influence the susceptibility of X-linked genes to transcriptional upregulation upon Xist loss

**Fig. 5:**
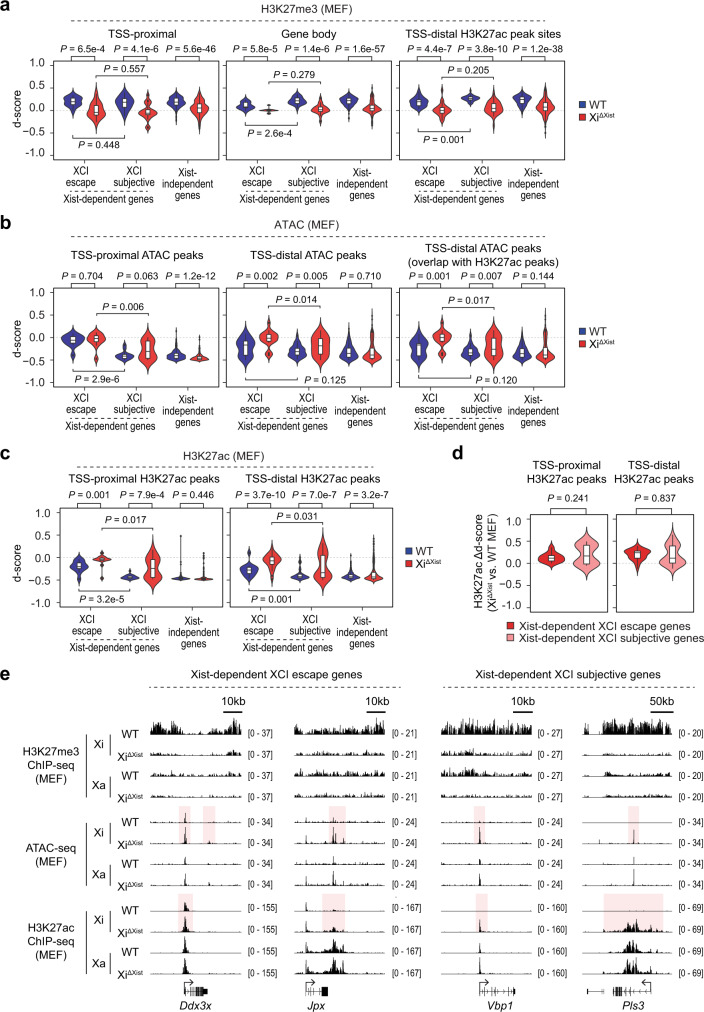
Association between changes in X-linked gene expression and chromatin structure in Xist-deficient MEFs. **a** Violin and box plots show H3K27me3 d-score within TSS-proximal, gene body and H3K27ac peak regions of the indicated Xist-dependent and Xist-independent gene sets in WT and Xi^ΔXist^ MEFs. **b** Violin and box plots show d-score of TSS-proximal and TSS-distal ATAC peaks, as well as TSS-distal ATAC peaks that show overlap with H3K27ac peaks for indicated Xist-dependent and Xist-independent gene sets in WT and Xi^ΔXist^ MEFs. **c** Violin and box plots show d-score of TSS-proximal and TSS-distal H3K27ac peaks of the indicated Xist-dependent and Xist-independent gene sets in WT and Xi^ΔXist^ MEFs. **d** Violin and box plots show change in d-score (Δd-score) of TSS-proximal and TSS-distal H3K27ac peaks of Xist-dependent XCI escape and subjective genes between Xi^ΔXist^ and WT MEFs. **e** Representative genome tracks show H3K27me3 ChIP-seq, H3K27ac ChIP-seq, and ATAC-seq signal across typical Xist-dependent XCI escape genes (e.g., *Ddx3x* and *Jpx*) and Xist-dependent XCI subjective genes (e.g., *Vbp1* and *Plt3*) on Xi and Xa in WT and Xi^ΔXist^ MEFs. Regions with increased H3K27ac deposition and chromatin accessibility on Xi are highlighted. See Supplementary Data 5 (**a**), 6 (**b**), and 7 (**a**–**d**) for list of genes and/or peaks in each group. Biological replicates of sequencing data (*n* = 2) were merged to maximize sequencing depth for allele-specific analysis (**a**–**d**). Boxplots are described in “Methods”. *P*-values were calculated using two-tailed unpaired Student’s *t*-test.

To further investigate the effect of H3K27me3 loss and increase in chromatin accessibility on the expression of XCI escape and XCI subjective genes, we performed H3K27me3 ChIP-seq and reanalyzed the previously published ATAC-seq data^40^ in WT and Xi^ΔXist^ MEFs. We observed significant reduction (*P* < 0.001) in H3K27me3 occupancy on the Xi at TSS-proximal and gene body regions of both Xist-dependent and Xist-independent genes in Xi^ΔXist^ MEFs (Fig. 5a, e and Supplementary Data 5). In addition, the d-score of ATAC peak intensity at TSS-distal regions was significantly increased for Xist-dependent XCI escape genes (e.g., *Ddx3x* and *Jpx*) (*P* = 0.002) and XCI subjective genes (e.g., *Pls3*) (*P* = 0.005) but not for Xist-independent genes (Fig. 5b, e and Supplementary Data 6). These results validated our findings in Lin− cells showing that Xist deletion is causal to H3K27me3 loss across the Xi and selective increase in chromatin accessibility at sites that associate with transcriptionally upregulated genes. Notably, because of the low basal level of H3K27me3 occupancy across the XCI escape genes on Xi in WT MEFs, the reduction in H3K27me3 d-score within gene bodies of Xist-dependent XCI escape genes (e.g., *Ddx3x* and *Jpx*) was milder than that of Xist-dependent XCI subjective genes (e.g., *Vbp1* and *Pls3*) (Fig. 5a, e). Similarly, because of the high chromatin accessibility around TSS of XCI escape genes in WT MEFs (e.g., *Ddx3x* and *Jpx*), only Xist-dependent XCI subjective genes were associated with a slight increase (*P* = 0.063) in ATAC peak d-score at TSS-proximal regions (e.g., *Vbp1*) (Fig. 5b). Therefore, we concluded that low H3K27me3 occupancy and high chromatin accessibility of XCI escape genes at the basal state in WT cells predispose these genes to transcriptional upregulation upon Xist loss.

A recent study reported that transcriptional upregulation of X-linked genes in Xist-deficient human B lymphoblastoid cells (GM12878) is associated with increased Histone 3 Lysine 27 acetylation (H3K27ac)^21^, a histone modification known to associate with active enhancers^42^. Here we performed H3K27ac ChIP-seq in MEFs and detected significantly increased (*P* ≤ 0.001) d-score for H3K27ac peaks associated with both TSS-proximal and TSS-distal regions of Xist-dependent genes but not Xist-independent genes (Fig. 5c, e and Supplementary Data 7). In addition, the TSS-distal H3K27ac deposition regions exhibited similar pattern of H3K27me3 loss and increased chromatin accessibility as the gene body regions and TSS-distal ATAC peak sites, respectively (Fig. 5a, b, e and Supplementary Data 7). However, no significant difference in the degree of H3K27ac increase was detected between Xist-dependent XCI escape genes and subjective genes, even though the former were associated with significantly higher (*P* ≤ 0.001) basal level of H3K27ac in WT MEFs (Fig. 5c, d and Supplementary Data 7). These results suggested that increased H3K27ac level does not directly contribute to the difference in the susceptibility of XCI escape genes and subjective genes to transcriptional upregulation upon Xist loss.

### Xist loss leads to increased YY1 binding at *cis*-regulatory elements of X-linked genes

Studies have shown that more than 90% of transcription factor (TF) binding events take place within open chromatin regions^43^. We found X-linked ATAC peaks with increased d-score in MEFs are uniquely enriched in binding motifs of 13 TFs, including YY1 and CTCF (Supplementary Fig. 8a and Supplementary Data 7). Among them, YY1 motif also showed relative enrichment in increased TSS-distal ATAC peak sites on the X chromosome compared to autosomes in both LSK+ and LSK− cells (Supplementary Fig. 8b and Supplementary Data 3). YY1 is a ubiquitous TF that can bind to and mediate interactions of *cis*-regulatory elements, including promoters and enhancers^44,45^. Previous studies have shown that YY1 is involved in XCI by regulating Xist expression and nucleation on the X chromosome^46–49^. However, the significance of Xist on Xi-specific YY1 binding has not been elucidated.

**Fig. 6:**
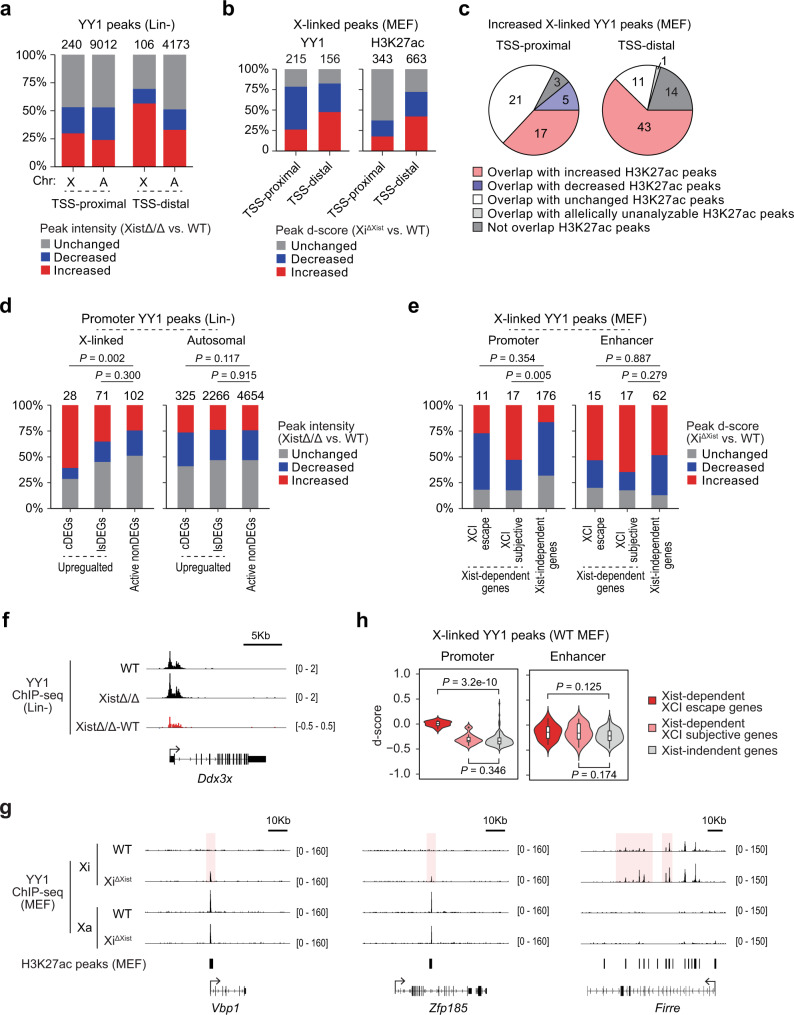
Xist deletion leads to increase in YY1 binding at the *cis*-regulatory elements on the Xi. **a** Bar graphs show percentages of increased, decreased, and unchanged YY1 peaks at TSS-proximal and TSS-distal regions on the X chromosome and autosomes in Lin− cells. **b** Bar graphs show percentages of increased, decreased, and unchanged X-linked YY1 (*left*) and H3K27ac (*right*) peaks at TSS-proximal and TSS-distal regions in MEFs. **c** Pie graphs show the number of increased YY1 peaks that overlap with increased, decreased, or unhanged H3K27ac peaks at TSS-proximal or TSS-distal regions of the X chromosome. **d** Bar graphs show comparison of the percentages of increased, decreased, and unchanged YY1 peaks at promoters of upregulated cDEGs and lsDEGs to nonDEGs on the X chromosome and autosomes in Lin− cells. **e** Bar graphs show comparison of the percentages of increased, decreased, and unchanged YY1 peaks at promoters and enhancers of the indicated gene sets on the X chromosome in MEFs. **f** Representative genome tracks show YY1 ChIP-seq signal across typical X-linked upregulated cDEGs associated with increased promoter YY1 binding (e.g., *Ddx3x*) in Lin− cells. **g** Representative genome tracks show YY1 ChIP-seq signal across typical Xist-dependent XCI subjective genes within increased YY1 binding at promoter (e.g., *Vbp1*) and enhancer (e.g., *Zfp18*) regions in MEFs upon Xist depletion. *Firre* exemplifies a typical Xist-independent gene associated with increased enhancer YY1 binding. **h** Violin and box plots show comparison of d-scores of promoter and enhancer YY1 peaks of indicated gene sets in WT MEFs. Analyzed peak numbers are indicated above each bar graph (**a**, **b**, **d**, **e**). See Supplementary Data 9 for a list of peaks for each group (**h**). Biological replicates of sequencing data (*n* ≥ 2) were merged to maximize sequencing depth for allele-specific analysis (**b**, **c**, **e**, **h**). Boxplots are described in “Methods”. *P*-values were calculated using two-tailed *Chi-squared* test (**d**, when peak number >300), two-tailed Fisher’s exact test (**d**, **e**), or two-tailed unpaired Student’s *t*-test (**h**).

To investigate how Xist deletion changes YY1 binding, we performed YY1 ChIP-seq in WT and XistΔ/Δ Lin− cells. We did not detect any difference in the overall YY1 binding or total number of YY1 peaks between WT and XistΔ/Δ Lin− cells (Supplementary Fig. 9 and Supplementary Data 8). However, consistent with the results of our motif analysis, we found higher percentage of increased YY1 peaks at the TSS-distal regions on the X chromosome compared to the autosomes (Fig. 6a). To elucidate how Xist deletion influences YY1 binding at promoters and enhancers on the Xi, we performed YY1 ChIP-seq in WT and Xi^ΔXist^ MEFs and compared the sites of differential YY1 peaks and H3K27ac peaks (Fig. 6b and Supplementary Data 7, 9). We found that increased YY1 peaks highly overlapped with increased H3K27ac peaks at the TSS-distal regions (Fig. 6c), suggesting a positive correlation between increased YY1 binding and increased enhancer activity at these regions. In contrast, increased YY1 peaks at TSS-proximal regions showed equally high overlap with both increased and unchanged H3K27ac peaks (Fig. 6c), pointing to a dual role of YY1 in regulating promoter and enhancer activity at the TSS-proximal regions.

An earlier study predicted the median distance between promoter and enhancer interactions to be ~125 kb^50^. Based on this criterion, we defined YY1 peaks located within ±3 kb from TSSs as promoter YY1 binding sites, while YY1 peaks that overlap with H3K27ac peaks and located within 3 to 125 kb from TSSs as enhancer YY1 binding sites (Supplementary Data 8 and 9). In Lin− cells, we observed enrichment of increased YY1 binding at promoters of upregulated cDEGs compared to active nonDEGs on the X chromosome (e.g., *Ddx3x*) but not on autosomes (Fig. 6d, f). Using MEFs, we further showed that it is the Xist-dependent XCI subjective genes (e.g., *Vbp1*) that exhibited increased YY1 binding at promoters upon Xist loss (Fig. 6e, g). In contrast, both Xist-dependent (e.g., *Zfp185*) and Xist-independent (e.g., *Firre*) genes exhibited high percentage of increased YY1 binding at the enhancers (Fig. 6e, g). These results suggested that Xist deletion leads to an increase in promoter YY1 binding specifically for XCI subjective genes, while the increase in enhancer YY1 binding is random on the Xi. Notably, the d-score of YY1 peaks at Xist-dependent XCI escape gene promoters was significantly higher than the other X-linked genes in WT MEFs (Fig. 6h) —a distribution similar to the pattern of basal chromatin accessibility (Fig. 5b). This data suggests that Xist represses YY1 binding on Xi by restricting chromatin accessibility. Based on this finding, we propose that the lack of increase in YY1 binding at promoters of XCI escape genes upon Xist-loss might be a result of their high basal level of chromatin accessibility that allows for preexisting YY1 activity in WT cells.

## Discussion

**Fig. 7:**
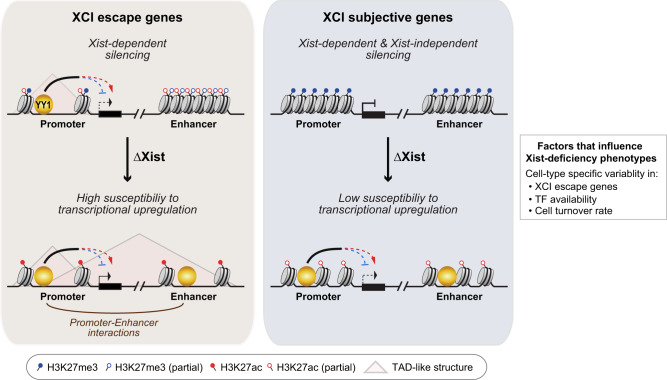
Working model on the regulatory role of Xist during XCI maintenance. Transcription of X-linked genes during XCI maintenance is regulated by Xist-dependent and Xist-independent mechanisms. Xist is required for maintaining H3K27me3 occupancy across Xi and limiting chromatin accessibility and H3K27ac deposition surrounding Xist-dependent genes. Low H3K27me3 occupancy and high chromatin accessibility at the basal state predispose XCI escape genes to be highly susceptible to transcriptional upregulation upon Xist loss. Increased chromatin accessibility on the Xi allows for higher binding frequency for transcriptional factors, such as YY1. While promoter-specific YY1 binding either activates or represses transcription, enhancer-specific YY1 binding might facilitate transcription through regulation of promoter–enhancer interactions within TAD-like structures at regions harboring XCI escape genes in Xist-deficient cells. Our findings support a model in which Xist-deficiency phenotypes in different cell lineages or tissues are influenced by cell type-specific characteristics including variability in types of XCI escape genes, cell turnover rate and availability of TFs.

Initiation of XCI in the developing embryo relies on Xist, which is continuously expressed in somatic cells during XCI maintenance. Here we investigated the role of Xist during XCI maintenance by utilizing hematopoietic-specific Xist knockout mouse model and clonal female MEF cell lines. We provided direct evidence supporting that Xist deletion leads to preferential transcriptional upregulation of XCI escape genes. Xist-deficient cells showed chromosome-wide loss of H3K27me3 occupancy and selective increase in chromatin accessibility and H3K27ac deposition surrounding transcriptionally upregulated X-linked genes (Fig. 7). Importantly, we found that XCI escape genes, which are associated with low H3K27me3 occupancy and high chromatin accessibility at the basal state in WT cells, exhibited milder decrease in H3K27me3 occupancy and milder increase in chromatin accessibility compared to XCI subjective genes in Xist-deficient cells (Fig. 7). Based on these findings, we concluded that Xist exerts gene-specific role of transcriptional repression on the Xi through a multi-layered epigenetic mechanism. Notably, Xist loss resulted in changes in Xi-specific H3K27me3 and H3K27ac occupancy and chromatin accessibility surrounding Xist-dependent XCI escape genes to Xa-specific levels (d-score = 0) (Fig. 5). In contrast, Xist-dependent XCI subjective genes continued to exhibit incomplete chromatin accessibility and H3K27me3 occupancy on the Xi in Xist-deficient cells (Fig. 5). These data indicate that the expression of Xist-dependent XCI subjective genes is repressed by both Xist-dependent and Xist-independent mechanisms during XCI maintenance (Fig. 7).

Our study also showed that Xist is a negative regulator of YY1 binding on the Xi by restricting chromatin accessibility (Fig. 7). Earlier studies have reported that Xist deletion causes reestablishment of TAD-like structures at regions harboring XCI escape genes in MEFs^39,40^. This reorganization could facilitate establishment of YY1-mediated promoter–enhancer interactions that favor gene expression and therefore contributes to the high susceptibility of XCI escape genes to transcriptional upregulation in Xist-deficient cells (Fig. 7). However, we cannot rule out the possibility that YY1 acts as a canonical TF at the promoters on the Xi by either activating or repressing transcription^44^. This might explain why we observed variable or no change in the allelic and overall expression of Xist-dependent genes in YY1 knockdown Xist-deficient MEFs (Supplementary Fig. 10 and Supplementary Data 10). These possibilities necessitate future structural studies involving YY1 HiChIP or ChIA-PET experiments to identify YY1 binding sites related to promoter-enhancers interactions on the Xi, and subsequent site-specific depletion of YY1 binding on the Xi to determine gene-specific function of YY1 in regulating transcription. Interestingly, we showed that Xist deletion leads to increase in YY1 binding at the enhancers within the X-linked *Firre* locus (Fig. 6g and Supplementary Fig. 11a). *Firre* gene is known to escape XCI in many mouse tissues^51^. In our study, *Firre* was defined as an Xist-independent gene in MEFs because it is not expressed in either WT or Xist-deficient MEFs. In contrast, *Firre* is expressed in Lin− cells and we detected elevated expression of specific *Firre* splice isoforms in Xist-deficient Lin− cells (Supplementary Fig. 11). This finding suggests potential involvement of other TFs that could influence X-linked gene transcription in a cell type-specific manner (Fig. 7).

Towards determining the functional role of Xist during XCI maintenance, our findings showed that Xist loss leads to lineage-specific differentiation and cell cycle defects in HSPCs, which could be a result of both primary effect of Xist loss and secondary effect of systematic hematopoietic stress that accumulates over time (Fig. 2). An earlier study has reported that stressing Xist-deficient polyp cells by carcinogen treatment induces cell turnover and leads to tumorigenesis—a phenotype that is not observed under normal conditions^28^. High number of blood cells turn over per day^52^. We propose that this nature of blood cells facilitates accumulation of systematic hematopoietic stress in Xist-deficient female mice, providing an explanation to the high sensitivity of hematopoietic system to Xist loss (Fig. 7). On the other hand, we found that XCI escape genes showing high susceptibility to transcriptional upregulation upon Xist loss is a tissue- and cell type-independent feature (Fig. 3e). We thus conject that increased expression of XCI escape genes is a primary effect of Xist loss and likely acts as the initial trigger for development of hematopoietic malignancies in Xist-deficient female mice.

Studies have shown that the landscape of XCI escape genes is highly variable between cell types^51^. We found that several cell cycle- and immune-related DEGs, including *Klf4*, *Flna*, and *Tlr7*, have been previously reported to escape XCI in mouse^51,53^ and were only transcriptionally upregulated in LSK+ cells but not in LSK− cells upon Xist loss (Fig. 3c). This argues that the difference in the cell type-specific expression of XCI escape genes likely contributes to the variability in Xist-deficiency phenotypes in different cell types^18–22^ (Fig. 7). However, due to the lack of SNPs to perform allele-specific analysis, we were not able to map the correlation between XCI escape genes and transcriptional upregulated genes in different HSPC populations using our current mouse model. This limitation highlights the need to generate hybrid mouse models carrying high-density SNPs. These mouse models can be utilized to address the role of XCI escape genes during the development of hematopoietic malignancies by targeting expression of escape genes on the Xi in Xist-deficient cells to a level comparable to the level in WT cells. In addition, while MEFs used in our study are compatible for revealing common Xist-mediated mechanisms during XCI maintenance, future studies involving the use of hybrid mouse models would be instrumental for dissecting mechanisms that underlie cell type-specific roles of Xist during hematopoiesis.

## Methods

### Mice and cell lines

Xist^*2lox/2lox*^ mice (129Sv4/Jae strain) and B6.Cg-*Commd10*^*Tg (Vav1-cre) A2Kio*^/J (Vav-iCre, Jax 008610, C57BL/6J stain) mice were obtained from the Mutant Mouse Resource and Research Center (MMRRC) at the University of North Carolina (UNC) and The Jackson Laboratory, respectively. Breeding and genotyping of WT, XistΔ/+, and XistΔ/Δ mice were performed following a previously published protocol^18^. Experiments were performed using 3–5 months old female mice. Mouse husbandry and experiments were carried out as stipulated by the Duke University Institutional Animal Care and Use Committee (IACUC). Mouse housing conditions were in a 12-h light-dark cycle at 72 +/− 2 °F with 40–70% humidity with *ad libitum* access to food and filtered water. Environmental enrichment, such as mouse houses and nesting material were provided to all animals. WT and Xi^ΔXist^ MEFs (gift from Lee lab) were cultured in complete DMEM media containing 10% fetal bovine serum (FBS) (Sigma F2442), 10 mM HEPES, 0.1 mM MEM Non-Essential Amino Acids (NEAA), 1% Penicillin/Streptomycin, and 0.2% 2-Mercaptoethanol.

### shRNA transduction of MEF cells

To knockdown YY1 expression, Xi^ΔXist^ MEFs were transduced using control (scramble) or mouse YY1-specific shRNA lentivirus particles^54^. Briefly, TRC2-pLKO.5-puro vectors carrying scramble (shSCR (Sigma, SHC202)) or YY1-specific (shYY1-1 (Sigma, TRCN0000311794, Target sequence: CCCTAAGCAACTGGCAGAATT) and shYY1-2 (Sigma, TRCN0000312837, Target sequence: ACATTGCTAAGATGCTATATC)) shRNA sequences were used to generate lentivirus particles in HEK293T cells. Following viral transduction for 24 h, cells were selected in media containing 2 μg/ml of Puromycin for 48–72 h. Next, cells were recovered in fresh growth media for 24 h followed by RNA extraction using TRIzol reagent (Invitrogen) and confirmation of knockdown by RT-qPCR.

### Total RNA extraction, reverse transcription, and real-time PCR

Total RNA from Lin− cells (200 × 10^3^) or MEFs (3 × 10^6^) were isolated using TRIzol reagent (Invitrogen, 15596026) according to the manufacturer’s instructions. To isolate total RNA from LSK+ (30 × 10^3^) and LSK− (100 × 10^3^) cells, RNeasy Plus Micro kit (Qiagen, 74034) was utilized. For reverse transcription, cDNA was prepared using M-MLV Reverse Transcriptase (Invitrogen, 28025013) with random hexamers (Invitrogen, 48190011). Real-time PCR (qPCR) was performed using iTaq Universal SYBR Green Supermix (Bio-Rad, 172-5124) with the following primer sets. *Gapdh*: (5′-ATG AAT ACG GCT ACA GCA ACA GG-3′; 5′-GAG ATG CTC AGT GTT GGG GG-3′); *Yy1*: (5′-CGA CGGT TGT AAT AAG AAG TTT G-3′; 5′-ATG TCC CTT AAG TGT GTA G-3′); *Firre* 5′ transcript: (5′-CAA ATT CAA GCA GGC AAG GG-3′; 5-AGG TAT GCT TCA CCT CTC CT-3′)^55^; *Firre* mid transcript: (5′-TTC CTC ATT CCC CTT CTC CT-3′; 5′-ACC AGG TAC CGT GAG CAA TC-3′)^56^; *Firre* 3′ transcript: (5′-TGT CCA TCC TTA TCC AGG TGC-3′; 5′-TGT GGG CAC CCA AGT CAT TA-3′); *Firre* full transcript: (5′-AAC AGT GCC CAT TTC AGT CC-3′; 5′-TTT TTC ATG CAG GGT GAT TG-3′)^55^. Relative gene expression was calculated by the standard curve method and normalized to *Gapdh* mRNA level.

### Isolation of bone marrow cells

Mouse bones (tibias and femurs) were dissected, and bone marrow (BM) was flushed with 1xPBS supplemented with 2% FBS (2% F/PBS) into a 50-ml tube (Falcon) using a 22.5-gauge needle. Cell suspension was pipetted up and down for several times and filtered through a 70 μm cell strainer (Falcon, 352350) to obtain a single-cell suspension. Red blood cells in the cell suspension were lysed by ACK Lysing buffer (Gibco, A10492-01) according to the manufacturer’s instructions.

### FACS

To label lineage-positive cells, the following antibody cocktail containing biotinylated antibodies against mouse CD11b (BD, 553309, M1/70 clone; 1:200), CD3e (BD, 553060, 145-2C11 clone; 1:200), CD45R/B220 (BD, 553088, RA3-6B2 clone; 1:200), CD8a (BD, 553029, 53-6.7 clone; 1:200), CD4 (BD, 553728, GK1.5 clone; 1:200), Ly-6C/G (BD, 553125, RB6-8C5 clone; 1:200) and TER-119 (BD, 553672; 1:200) in combination with BV421-conjugated Streptavidin (BD, 563259; 1:200) was used. To distinguish between LSK+ and LSK− cells, biotinylated lineage-specific antibody cocktail was used in combination with FITC-conjugated Ly-6A/E (Sca-1) (BD, 557405, D7 clone; 1:100) and PE-conjugated CD117 (c-Kit) (BioLegend, 105808, 2B8 clone; 1:100) antibodies. To distinguish LSK+ subpopulations (HSC, MPP HPC1, HPC2), cells were further labeled with PE/Cy7-conjugated CD150 (BioLegend, 115914, TC15-12F12.2 clone; 1:100) and APC/Cy7-conjugated CD48 (BioLegend, 103432, HM48-1 clone; 1:100) antibodies. To distinguish LSK− subpopulations (MEP, GMP, CMP), cells were labeled with AlexaFluor647-conjugated CD34 (BD, 560230, RAM34 clone; 1:100) and PE/Cy7-conjugated CD16/32 (FcγR) (BD, 560829, 2.4G2 clone; 1:100) antibodies. To identify CLP, we stained cells with PE/Cy7-conjugated CD127 (IL7Rα) (BD, 560733, SB/199 clone; 1:100). 7-Amino-Actinomycin D (7-AAD) (BD, 559925; 1:200) solution was used to label the nonviable cells. Labeled cells were filtered through a 70 μm cell strainer before FACS analysis or sorting. FACS analysis was conducted on BD FACSCanto II Cell Analyzer (BD). Cell sorting was conducted on MoFlo Astrios Cell Sorter (BD) or MA900 Multi-Application Cell Sorter (Sony). Data were analyzed using FlowJo software (v10.8.1).

### Ki67 cell cycle assay

For Ki67 cell cycle assay, bone marrow cells were labeled using biotinylated lineage antibody cocktail in combination with PerCP-conjugated streptavidin (BD, 554064) and antibodies against Sca-1, c-Kit, CD150, and CD48 as described under FACS protocol above. Labeled cells were fixed and permeabilized using Fixation/Permeabilization solution (BD, 554714) at 4 °C for 20 min and washed with 1x BD Perm/Wash buffer (BD, 554714). Cells were next incubated in 1x BD Perm/Wash buffer containing AlexaFluor674-conjugated anti-Ki67 antibody (BioLegend, 652408, 16A8 clone; 1:100) or AlexaFluor674-conjugated anti-IgG2 antibody (BioLegend, 400526, RTK2758 clone; 1:100) at 4 °C overnight. The next day, cells were washed with 1X BD Perm/Wash buffer and resuspend in 2% FBS containing 1xPBS. DAPI solution (Thermo, 62248; 1:500) was used to stain DNA before FACS analysis.

### Preparation of metaphase chromosome spreads

Bone marrow cells from mice were labeled with a cocktail of biotin-conjugated lineage marker antibodies in combination with Streptavidin Particles Plus (BD, 557812) according to the manufacturer’s instructions. Lineage-positive cells were depleted using Cell Separation Magnet (BD, 552311) according to the manufacturer’s instructions to obtain lineage-negative (Lin−) bone marrow cells. To arrest cells at metaphase, ~2.5 × 10^6^ Lin− cells were incubated in complete RPMI media containing 100 ng/ml nocodazole (Sigma) at 37 °C for 2 h. Cells were then resuspended in 5 ml of 0.056 M KCl and incubated for 30 min at room temperature. KCl-treated cells were then resuspended in 1 ml of cold methanol: glacial acetic acid (3:1) solution and spun down at 1000 rpm for 10 min at 4 °C for fixation. The fixation step was repeated for three times, and every time 200 μl of solution was left when removing the supernatant. After the last fixation step, the metaphase cell suspension can be stored at −20 °C for at least 1 year. To prepare metaphase chromosome spreads, 20 μl of metaphase cell suspension was released onto a pre-chilled glass slide with the help of a pipette from 1.5-m height. The spreads were air dried and used for H3K27me3 IF staining and X-paint DNA FISH.

### Click-iT EdU assay

Sorted LSK+ cells were cultured in HSPC media following a published protocol with minor modifications^35^. Briefly, HSPC media is composed of StemSpan™ SFEM medium that was supplemented with mouse SCF (PeproTech, 250-03; 50 ng/ml), mouse TPO (PeproTech, 315-14; 50 ng/ml), mouse Flt3-Ligand (Flt-L) (PeproTech, 250-31L; 50 ng/ml) and human IL-11 (PeproTech, 200-11; 50 ng/ml) and 1% Penicillin/Streptomycin. Sorted LSK+ cells were washed twice using HSPC media, seeded onto a 96-well plate (10 × 10^3^ cells in 200 μl of HSPC media/well), and then cultured for 48 h at 37 °C with half volume media change every 24 h. Following 48-h of culture, EdU (ThermoFisher, C10338) was added to a final concentration of 10 μM. Next, LSK+ cells were transferred onto Poly-D-Lysine-coated 12-well glass slides (Fisher Scientific, 9991090) in HSPC medium and cultured for 1 h at 37 °C for EdU incorporation and cell attachment. The slides were then washed twice with ice-cold 1xPBS, permeabilized in ice-cold CSK solution (10 mM PIPES/KOH pH 8.6, 100 mM NaCl, 300 mM Sucrose, 1 mM MgCl_2_) containing 0.5% Triton X-100 for 10 min, fixed in 4% Paraformaldehyde (PFA) at room temperature for 10 min and stored in 70% EtOH at 4 °C until use. Prepared slides were first utilized for Xist RNA FISH, followed by a fixation step in 4% PFA/1xPBS for 5 min at room temperature. The slides were washed three-times with 3% BSA/1xPBS and Click-iT reaction was performed following manufacturer’s instructions (ThermoFisher, C10338). After ClickiT reaction, the slides were washed three times with 3% BSA/1XPBS before H3K27me3 IF staining using anti-H3K27me3 primary antibody (Cell Signaling, 9733, C36B11 clone, 1:200) and AlexaFluor555-conjugated goat anti-rabbit IgG secondary antibody (Invitrogen, A32732, 1:500).

### RNA FISH, DNA FISH, and IF staining

RNA FISH of LSK+ cells was performed following a published protocol with minor modifications^24^. Single-stranded Xist riboprobe was generated by in vitro transcription of Xist plasmid (E6, gift from Lee lab) that contains the intronic sequence between Xist exon 7 and 8 using biotin-16-UTP (Roche, 11388908910) and MAXIscript SP6 kit (Invitrogen, AM1322). The RNA probe is diluted in hybridization buffer (50% Formamide, 2xSSC, 2 mg/ml BSA, 10% Dextran sulfate-500k) at a concentration of 10 ng/μl, denatured at 75 °C for 10 min, and pre-annealed at 42 °C for 30–90 min before hybridization. To perform RNA FISH, 10–50 × 10^3^ LSK+ cells were incubated on each well of a 12-well glass slide (Fisher Scientific) at 37 °C for 30 to 60 min and processed as described above. The cells were then dehydrated in ice-cold 70%, 90%, and 100% EtOH sequentially for 2 min per step and air dried at room temperature for 5 min. Approximately, 5–6 μl of denatured and pre-annealed Xist RNA probe (~100 ng) was added onto each well of the slide. The slide was then covered with a clean coverslip, sealed with rubber cement, and incubated overnight at 37 °C in a dark humid chamber. Slides were then washed three times using 2xSSC containing 50% Formamide at 45 °C for 5 min followed by a single wash in 0.1xSSC at 45 °C for 5 min. Slides were quickly rinsed with 1xPBS containing 0.2% Tween-20 at room temperature for 10 s. To detect the biotin-labeled Xist RNA probe, the slides were washed in blocking solution (4xSSC, 1% BSA, 0.1% Tween-20) at 37 °C for 10 min, hybridized with AlexaFluor647-conjugated streptavidin (Invitrogen, S21374; 1:400 diluted in blocking solution) at 37 °C for 40 min in a dark humid chamber, and then washed three times in 4xSSC, 0.1% Tween-20 solution at 45 °C for 10 min each.

To perform sequential H3K27me3 or H3Ser10P immunostaining of LSK+ cells following RNA FISH, the slides were blocked with IF blocking solution (1xPBS, 1% BSA, 0.1% Tween-20) at room temperature for 1 h, incubated with primary antibodies including anti-H3Ser10P (Millipore, 05-806, 3H10 clone; 1:200) and anti-H3K27me3 (Cell Signaling, 9733, C36B11 clone; 1:200) diluted in IF blocking solution at room temperature for 1 h, washed for three times with 1xPBS, and then incubated with secondary antibodies for 1 h at room temperature. AlexaFluor555-conjugated goat anti-mouse IgG secondary antibody (Invitrogen, A21422; 1:500) was used to detect H3Ser10P. AlexaFluor488-conjugated (Invitrogen, A11008; 1:500) or AlexaFluor555-conjugated (Invitrogen, A32732; 1:500) goat anti-rabbit IgG secondary antibody was used to detect H3K27me3. The slides were washed for three times in 1x PBS at room temperature before mounting and imaging.

For DNA FISH using metaphase chromosome spreads, FITC labeled mouse X-paint (Applied Spectral Imaging) was used. To prepare X-paint probe, 10 μl of X-paint solution was dried together with 10 μg of mouse Cot-1 DNA (Invitrogen, 18440-016) using Savant Integrated SpeedVac Concentrator and the pellet was suspended using 50 μl (5x volume) of hybridization buffer. The X-paint probe was denatured at 80 °C for 10 min and pre-annealed at 37 °C for 30–90 min before use. To perform sequential H3K27me3 IF staining and X-paint DNA FISH, air dried metaphase chromosome spread slides were first blocked in IF blocking solution for 1 h at 37 °C, followed by incubation with anti-H3K27me2/me3 antibody (Active Motif, 39535, 7B11 clone; 1:200) for 3 h at 37 °C. Slides were then washed three times in 1xPBS solution at room temperature and then hybridized with AlexaFluor555-conjugated goat anti-mouse IgG secondary antibody (Invitrogen, A21422; 1:500) for 1 h at 37 °C. After washing three times with 1XPBS, spreads were re-fixed with 4% PFA for 10 min at room temperature, denatured in 70% Formamide/2xSSC buffer at 80 °C for 10 min, dehydrated in ice-cold 70%, 90%, and 100% EtOH sequentially for 2 min per step, and then air dried at room temperature for 5 min. Approximately, 10 μl of denatured and pre-annealed X-paint probe was added onto each slide. Probe solution was covered using a clean 18 mm × 50 mm coverslip, sealed with rubber cement, and then incubated overnight at 37 °C in a dark humid chamber overnight. Slides were then washed three times in 50% Formamide/2xSSC at 45 °C for 5 min, three times in 2xSSC at 45 °C for 5 min, and once in 0.1xSSC at 45 °C for 5 min.

As a last step of FISH and IF staining, air-dried slides were mounted using Vectashield containing DAPI. A coverslip was placed on the slide and sealed with nail polish before imaging. Images were acquired on a Leica Automated Upright Microscope (Leica, DM5500B) using a Leica DFC365 FX CCD camera and analyzed using Leica Application Suite X and FiJi software (v2.3.0/1.53f).

### RNA-seq

Before library preparation, RNA quality was assessed using 2100 Bioanalyzer (Agilent Technologies). Only RNA samples with high integrity (RNA integrity number (RIN) > 8.5) were used for RNA-seq library preparation. RNA-seq libraries of Lin− cells were prepared using Ovation RNA-Seq System Kit (NuGen, 0403-32 and 0348-32). LSK+ and LSK− RNA-seq libraries were prepared using SMART-Seq v4 Ultra Low Input RNA Kit (Takara, 634888) together with KAPA HyperPrep kit (Roche, 07962347001). MEF RNA-seq libraries were prepared using KAPA RNA HyperPrep kit (Roche, 8098093702). Sequencing type, platform, and depth for all RNA-seq samples are listed in Supplementary Data 11.

### ATAC-seq

ATAC experiments were performed following a published protocol with minor modifications^41^. Briefly, bone marrow LSK+ or LSK− cells (~10 × 10^3^) were lysed in ice-cold lysis buffer (10 mM Tris-HCl, pH 7.4, 10 mM NaCl, 3 mM MgCl_2_, 0.1% NP-40) for 5 min and then washed twice with 10 mM Tris-HCl, pH 7.4. In the second wash, 25 μl of lysate was left when supernatant was removed and mixed with 25 μl of 2XTD buffer (66 mM Tris-Ac pH 7.8, 132 mM KAc, 20 mM MgAc, 32% Dimethyl Formamide) containing 1 μl of Tn5 with loaded tagmentation adapters. Transposition reaction mixture was incubated using a thermocycler rotating at 500 rpm for 1 h at 37 °C. Tagmented DNA fragments were purified using MinElute PCR Cleanup Kit (Qiagen, 28206) and amplified for 15 PCR cycles using Q5 polymerase (NEB, M0491). PCR products were run on a gel, and fragments of 200 bp to 800 bp were isolated and sequenced. Sequencing type, platform, and depth for all ATAC-seq samples are listed in Supplementary Data 11.

### ChIP-seq

H3K27me3 and H3K4me3 ChIP experiments were performed following a published protocol with minor changes^57^. Briefly, MEF or Lin− cells (1.5–3 × 10^6^) were cross-linked with 1% formaldehyde for 10 min at room temperature and the reaction was stopped by adding glycine (final concentration, 125 mM) and incubation for 5 min at room temperature. Cross-linked cells were treated with hypotonic buffer (10 mM HEPES-NaOH, pH 7.9, 1.5 mM MgCl_2_, 10 mM KCl, 0.2% NP-40, 1 mM DTT, 1 mM PMSF, 1x protease inhibitor cocktail (Sigma, P8340)) at 4 °C for 10 min, lysed in 150  μl of ChIP lysis buffer (50 mM Tris-HCl, pH 7.9, 10 mM EDTA pH 8.0, 1% SDS, 1 mM PMSF, protease inhibitor cocktail (Sigma, P8340)) on ice for 10 min, and sonicated for 35 cycles (30″ ON + 30″ OFF) using Diagenode Bioruptor to shear the chromatin into 200–1000 bp fragments. The lysate was diluted with 9x volumes of ChIP dilution buffer (16.7 mM Tris-HCl, pH 7.9, 167 mM NaCl, 1.2 mM EDTA pH 8.0, 1.1% Triton X-100, 1 mM PMSF). The fragmented chromatin was pre-cleared using 15 μl of pre-washed Protein A/G agarose beads (ThermoFisher, 20423) for 1 h at 4 °C. The pre-cleared chromatin fragments were equally separated into two aliquots. Each aliquot was incubated with either 2 μl of anti-H3K27me3 (Abcam, ab6002) or 2 μl of anti-H3K4me3 (Abcam, ab8580) antibody at 4 °C overnight with rotation, followed by incubation with pre-washed Protein A/G agarose beads (Thermo Scientific, 20423) for 1–2 h at 4 °C. The chromatin-agarose complexes were washed at 4 °C with the following buffers: low salt wash buffer (20 mM Tris-HCl, pH 7.9, 100 mM NaCl, 2 mM EDTA pH 8.0, 0.1% SDS, 1% Triton X-100) for 10 min, high salt wash buffer (20 mM Tris-HCl, pH 7.9, 500 mM NaCl, 2 mM EDTA pH 8.0, 0.1% SDS, 1% Triton X-100) for 10 min, LiCl buffer (10 mM Tris-HCl, pH 7.9, 250 mM LiCl, 1% NP-40, 1% sodium deoxycholate, 1 mM EDTA pH 8.0) for 10 min, and twice with TE buffer for 5 min. The chromatin was eluted by incubating the beads in elution buffer (1% SDS, 0.1 M NaHCO_3_) for 15 min at room temperature.

For YY1 and H3K27ac ChIP, MEF or Lin− cells (1.5–3 × 10^6^) were cross-linked with 1% formaldehyde for 10 min (H3K27ac) or 15 min (YY1) at RT and the reaction was stopped by glycine as described above. Cross-linked cells were treated with cell lysis buffer (10 mM Tris-HCl pH 7.5, 10 mM NaCl, 3 mM MgCl_2_, 0.5% NP-40, 1 mM DTT, 1 mM PMSF, 1x protease inhibitor cocktail (Sigma, P8340)) for 10 min at 4 °C, washed twice with MNase reaction buffer (10 mM Tris-HCl pH 7.5, 10 mM NaCl, 3 mM MgCl_2_, 1 mM CaCl_2_, 0.2% NP-40, 1 mM DTT, 1 mM PMSF, 1x protease inhibitor cocktail), resuspended in 200 μl of MNase reaction buffer containing 2.5U of MNase (NEB, M0247) and incubated for 10 min at 37 °C to digest chromatin into <1000 bp fragments. MNase digestion was stopped by adding 20 μl of 0.5 M EDTA pH 8.0 into the reaction. The lysate was sonicated for 3 cycles (10″ ON + 20″ OFF) using Microson ultrasonic cell disruptor (Misonix) to break up nuclear membrane followed by spinning at 21,000 × *g* for 10 min at 4 °C. The supernatant containing fragmented chromatin was incubated with 2 μl of anti-YY1 (Cell Signaling, 46395) or 2 μl of anti-H3K27ac (Abcam, ab4729) antibody overnight at 4 °C, followed by incubation with 10 μl of pre-washed Protein G Magnetic Beads (Invitrogen, 10003D) for 1–2 h at 4 °C. Chromatin-bead complexes were sequentially washed with low salt wash buffer, high salt wash buffer, LiCl buffer and TE buffer as described above, and the chromatin was eluted in elution buffer by incubating at 65 °C for 40 min.

Eluted chromatin was reverse cross-linked by incubating in 200 mM of NaCl at 65 °C overnight followed by Proteinase K (20 μg/ml) treatment for 2 h at 55 °C. DNA was purified using MinElute PCR purification kit (Qiagen, 28006) and quantified using the fluorometric quantitation Qubit 2.0 system (Thermo Fisher Scientific). ChIP-seq libraries were prepared using KAPA HyperPrep kit (Roche, 07962347001). Sequencing type, platform, and depth for all ChIP-seq samples are listed in Supplementary Data 11.

### RNA-seq data analysis

For total gene expression analyses, RNA-seq reads were trimmed using TrimGalore (v0.6.0, -q 15)^58^ and then aligned to mm10 reference genome by TopHat (v2.1.1, --b2-very-sensitive --no-coverage-search)^59^. Reads with low alignment quality (MAPQ < 30) were filtered out using Samtools (v1.10, -q 30)^60^. Aligned reads for each gene were counted by featureCounts (v1.6.3, -t exon -g gene_id)^61^ and normalized using DEseq2 (v1.30.1)^62^. Gene expression level was quantified using Fragments Per Kilobase of transcript per Million mapped reads (FPKM). Genes with FPKM ≥ 1 were defined as actively transcribed genes. Active genes showing consistent increase or decrease in read counts in both biological replicates were determined as differentially expressed genes (DEGs), while the others were determined as non-differentially expressed genes (nonDEGs). To compare Xist deletion-induced DEGs between tissues, RNA-seq data of other conditional Xist knockout mouse models were obtained from European Bioinformatics Institute’s repository under accession number E-MTAB-8161 (kidney, crypt, and polyp)^28^ and from GEO database under accession number GSE97077 (brain)^20^. Two biological replicates of published RNA-seq results of each tissue-specific Xist-mutant mouse model were used for analyses using the same methods as our HSPC RNA-seq data. Gene Ontology (GO) analysis was conducted using clusterProfiler R package (v3.18.1)^63^. Genes associated with GO terms containing keywords including “cell cycle”, “nuclear division”, “DNA replication”, “chromosome segregation”, “chromatid segregation”, and “chromatid separation” were considered as cell cycle-related genes. Genes associated with GO terms containing keywords including “immune”, “antigen”, “interferon”, and “defense response” were considered as immune-related genes. Gene set enrichment analyses (GSEA) was conducted using the GSEA software (v4.0.3) with MSigDB ontology gene sets (v6.2) or customized signature gene sets of myeloid progenitors^31,64^. To obtain signature genes for myeloid progenitors, published RNA-seq data for CMPs, GMPs, and MEPs (GSE60103)^65^ was utilized. Genes that show significant upregulated expression (*p* < 0.05 and FPKM ≥ 1) in one myeloid progenitor population and are not actively transcribed (FPKM < 1) in the other two myeloid progenitor populations were determined as signature genes.

For allele-specific expression analysis, RNA-seq data of MEFs was processed following steps described in an earlier publication^21^. Briefly, biological replicates of RNA-seq data were pooled to maximize sequencing depth. SNPs between 129S1/SvImJ and CAST/EiJ mouse strains were extracted from Welcome Sanger Mouse Genomes Project database (ftp://ftp-mouse.sanger.ac.uk). An N-masked mouse genome (in which all SNPs between 129S1/SvImJ and CAST/EiJ genome are replaced by “N”) was prepared by SNPsplit_genome_preparation function of SNPsplit package (v0.3.2)^66^. RNA-seq reads were aligned to the N-masked reference genome by TopHat (v2.1.1, --b2-very-sensitive --no-coverage-search). After alignment and removal of reads with low alignment quality (MAPQ < 30), the remaining aligned reads were split to 129S1/SvImJ or CAST/EiJ allele using SNPsplit. Allelically aligned reads for each gene were counted by featureCounts (v1.6.3, -t exon -g gene_id). X-linked genes with at least 10 allelically aligned reads were regarded as allelically analyzable. To describe the allele-specific expression status of each gene, an expression d-score was calculated using the formula:

1 $${\mbox{d-score}}={{{{{{\rm{Reads}}}}}}}_{{{{{{\rm{Xi}}}}}}}/({{{{{{\rm{Reads}}}}}}}_{{{{{{\rm{Xi}}}}}}}+{{{{{{\rm{Reads}}}}}}}_{{{{{{\rm{Xa}}}}}}})-0.5$$

where Reads_Xi_ and Reads_Xa_ are the number of reads aligned to 129Sv4/Jae X allele and CAST/EiJ X allele, respectively. Genes with Reads_Xi_ no less than 5% of Reads_Xa_ (Reads_Xi_/Reads_Xa_ ≥ 0.05) in WT MEFs were defined as XCI escape genes, while the other genes were defined as XCI subjective genes. When comparing expression d-scores between WT and Xi^ΔXist^ MEFs, only X-linked genes that are allelically analyzable in both WT and Xi^ΔXist^ MEFs were used for the analysis. Genes with Xi^ΔXist^ d-score − WT d-score ≥ 0.03 were referred as Xist-dependent genes, while the other genes were referred as Xist-independent genes. When comparing expression d-scores between Xi^ΔXist^ MEFs treated with shSCR and shYY1, only X-linked genes that are allelically analyzable in both shSCR and shYY1 treated MEFs were used for analysis. Genes with shYY1 d-score − shSCR d-score ≥ 0.03 were referred to as having increased expression d-score; genes with shSCR d-score − shYY1 d-score ≤ 0.03 were referred to as having decreased expression d-score; and the remaining genes were referred to as having unchanged expression d-score.

### ATAC-seq data analysis

For ATAC-seq in LSK+ and LSK− cells, reads were trimmed using TrimGalore and then aligned to mm10 reference genome by Bowtie2 (v2.3.5.1, --qc-filter --very-sensitive)^67^. Reads with alignment quality lower than 30 or reads aligned to mitochondrial DNA were removed using Samtools (v1.10). ATAC-seq results of all experimental replicates were merged to make bigwig files, metagene profiles, and heatmaps using deepTools (v3.5.0)^68^ with CPM normalization. ATAC-seq reads within TSS-proximal regions were counted by multicov function in bedtools (v2.25.0)^69^. The density of ATAC signals within TSS-proximal region of each gene was quantified by Reads Per Kilobase per Million mapped reads (RPKM):

2 $${{{{{\rm{RPKM}}}}}}=	{10}^{6}\,\times \,{{{{{\rm{Reads}}}}}}\,{{{{{\rm{count}}}}}}\,{{{{{\rm{within}}}}}}\,{{{{{\rm{region}}}}}}/({{{{{\rm{Total}}}}}}\,{{{{{\rm{reads}}}}}}\,{{{{{\rm{count}}}}}}\,\\ 	\times \,{{{{{\rm{Region}}}}}}\,{{{{{\rm{width}}}}}}\,({{{{{\rm{kb}}}}}}))$$

ATAC peaks were called using callpeak program (-q 0.01 -B -f BAMPE) in MACS2 (v2.2.7.1)^70^. Consensus ATAC peaks called in no less than 50% of samples were considered as real ATAC peaks. Reads within each ATAC peak site were counted (bUseSummarizeOverlaps = TRUE, summits = TRUE, filter = 0) and normalized (method = DBA_DESEQ2) using DiffBind R package (v3.0.13, https://bioconductor.org/packages/DiffBind/). ATAC peak intensity was measured by normalized Reads Per Kilobase (RPK):

3 $${{{{{\rm{RPK}}}}}}={{{{{\rm{Normalized}}}}}}\,{{{{{\rm{reads}}}}}}\,{{{{{\rm{count}}}}}}\,{{{{{\rm{within}}}}}}\,{{{{{\rm{peak}}}}}}/{{{{{\rm{Peak}}}}}}\,{{{{{\rm{width}}}}}}\,({{{{{\rm{kb}}}}}})$$

ATAC peaks that show consistent higher or lower intensity in XistΔ/Δ cells compared to WT cells in both biological replicates were defined as increased or decreased ATAC peaks, respectively.

For allele-specific analysis of published ATAC-seq data of MEFs (GSE109395)^40^, biological replicates of ATAC-seq data were pooled, aligned, and split into 129S1/SvImJ (Xi) or CAST/EiJ (Xa) genome using SNPsplit similar as described under allele-specific RNA-seq data analysis above. After alignment, duplicated reads were removed by MarkDuplicates tool of Picard (v2.0.1; http://broadinstitute.github.io/picard/). Bigwig files were prepared using deepTools without normalization. ATAC peaks called by MACS2 callpeak program (-q 0.01 -B -f BAMPE) in WT and Xi^ΔXist^ MEFs were merged using merge (-d 1000) function in bedtools. Allelically aligned reads within each peak site were counted by multicov function in bedtools. Peaks with at least 10 allelically aligned reads were considered as allelically analyzable and retained to calculate the d-score similar as allele-specific RNA-seq analysis. Peaks with increased (Xi^ΔXist^ d-score – WT d-score ≥ 0.03), decreased (Xi^ΔXist^ d-score – WT d-score ≤ −0.03), and unchanged d-score (−0.03 < Xi^ΔXist^ d-score – WT d-score < 0.03) were referred as allelically increased, decreased, or unchanged peaks, respectively.

ATAC peak annotation was performed using ChIPpeakAnno R package (v3.24.1)^71^. ATAC peaks within TSS-proximal regions were first annotated to the gene with the closest TSS. Next, TSS-distal ATAC peaks were annotated to the gene with the closest gene body region. Motif analysis with differential ATAC peaks was conducted using findMotifsGenome program (-size given -len 8,10 -mis 2 -h -mask) of HOMER (v4.11)^72^. Motifs with *p*-value < 0.01 and *q*-value < 0.01 were defined as significantly enriched motifs.

### ChIP-seq data analysis

For H3K27me3, H3K4me3, and YY1 ChIP-seq in Lin− cells, reads were trimmed using TrimGalore and then aligned to mm10 reference genome by Bowtie2 (v2.3.5.1, --qc-filter --very-sensitive). Reads with alignment quality lower than 30 were removed using Samtools (v1.10). Duplicated reads in YY1 ChIP-seq data were removed after alignment. ChIP-seq results of all biological replicates were merged to make bigwig files, metagene profiles and heatmaps using deepTools (v3.5.0) with CPM normalization. YY1 peaks were called using findPeaks (-region -size 500 -minDist 500 -F 0 -L 4 -C 2) program in HOMER, and consensus YY1 peaks called in no less than 50% of samples were considered as real YY1 peaks. Measurement of H3K27me3 and H3K4me3 density as well as YY1 peak intensity were performed using the same methods as ATAC-seq data analysis.

For allele-specific analysis of H3K27me3, YY1, and H3K27ac ChIP-seq data of MEFs, biological replicates of ChIP-seq data were pooled, aligned and split into 129S1/SvImJ (Xi) or CAST/EiJ (Xa) genome using SNPsplit similar as allele-specific RNA-seq data analysis. Duplicated reads were removed after alignment. Bigwig files were prepared using deepTools without normalization. YY1 and H3K27ac peaks in MEFs were both called using findPeaks (-region -size 500 -minDist 500 -F 0 -L 4 -C 2) program in HOMER. The following allele-specific ChIP-seq data analysis was performed using the same steps as allele-specific ATAC-seq data analysis.

H3K27ac peak annotation was performed following the same steps as ATAC peaks. For YY1 peak annotation, we first defined YY1 peaks located within ±3 kb from TSSs as promoter YY1 peaks. Next, we defined YY1 peaks that overlap with H3K27ac peaks and located within 3 to 125 kb from TSSs as enhancer YY1 peaks. We annotated promoter or enhancer YY1 peaks to all genes within ±3 kb or ±125 kb, respectively. Peaks sharing no less than 1 bp in their peak sites were considered as overlapping.

### Statistical analysis

Statistical tests used for calculating *p*-values are indicated at the end of legend of each figure. Exact *p*-values were indicated within each figure. For *p*-values that were too small to be represented within three decimal places, we utilized scientific notation format. *P* < 0.05 was considered significant if not specifically defined. For boxplots, 25–75 percentile was shown with median and outliers, and whiskers encompass 1.5× the interquartile range.

### Reporting summary

Further information on research design is available in the Nature Research Reporting Summary linked to this article.

## Supplementary information

Supplementary Information

Description of Additional Supplementary Files

Supplementary Data 1

Supplementary Data 2

Supplementary Data 3

Supplementary Data 4

Supplementary Data 5

Supplementary Data 6

Supplementary Data 7

Supplementary Data 8

Supplementary Data 9

Supplementary Data 10

Supplementary Data 11

Reporting Summary

## Data Availability

The data that support this study are available from the corresponding author upon reasonable request. Raw sequencing data generated in this study have been deposited in the Gene Expression Omnibus (GEO) under accession number GSE184776. RNA-seq data of other Xist knockout mouse models were obtained from European Bioinformatics Institute’s repository under accession number E-MTAB-8161 and from GEO database under accession number GSE97077. ATAC-seq data of MEFs were obtained from GEO database under accession number GSE109395. Source data are provided with this paper.

## Source data

Source Data File

## Code availability

The custom analysis pipelines for all genomic analyses are available from the corresponding author upon request with no restrictions.
