# Supplementary material for: Xist exerts gene-specific silencing during XCI maintenance and impacts lineage-specific cell differentiation and proliferation during hematopoiesis
Source: Nat Commun. 2022 Aug 1;13:4464. doi: 10.1038/s41467-022-32273-5 (PMC9343370; doi:10.1038/s41467-022-32273-5)
Supplement: Supplementary file 1 — Supplementary Information [file 41467_2022_32273_MOESM1_ESM.pdf]

**Supplementary Information**

**Xist exerts gene-specific silencing during XCI maintenance and impacts lineage-specific cell differentiation and proliferation during hematopoiesis**

Yang *et al.*

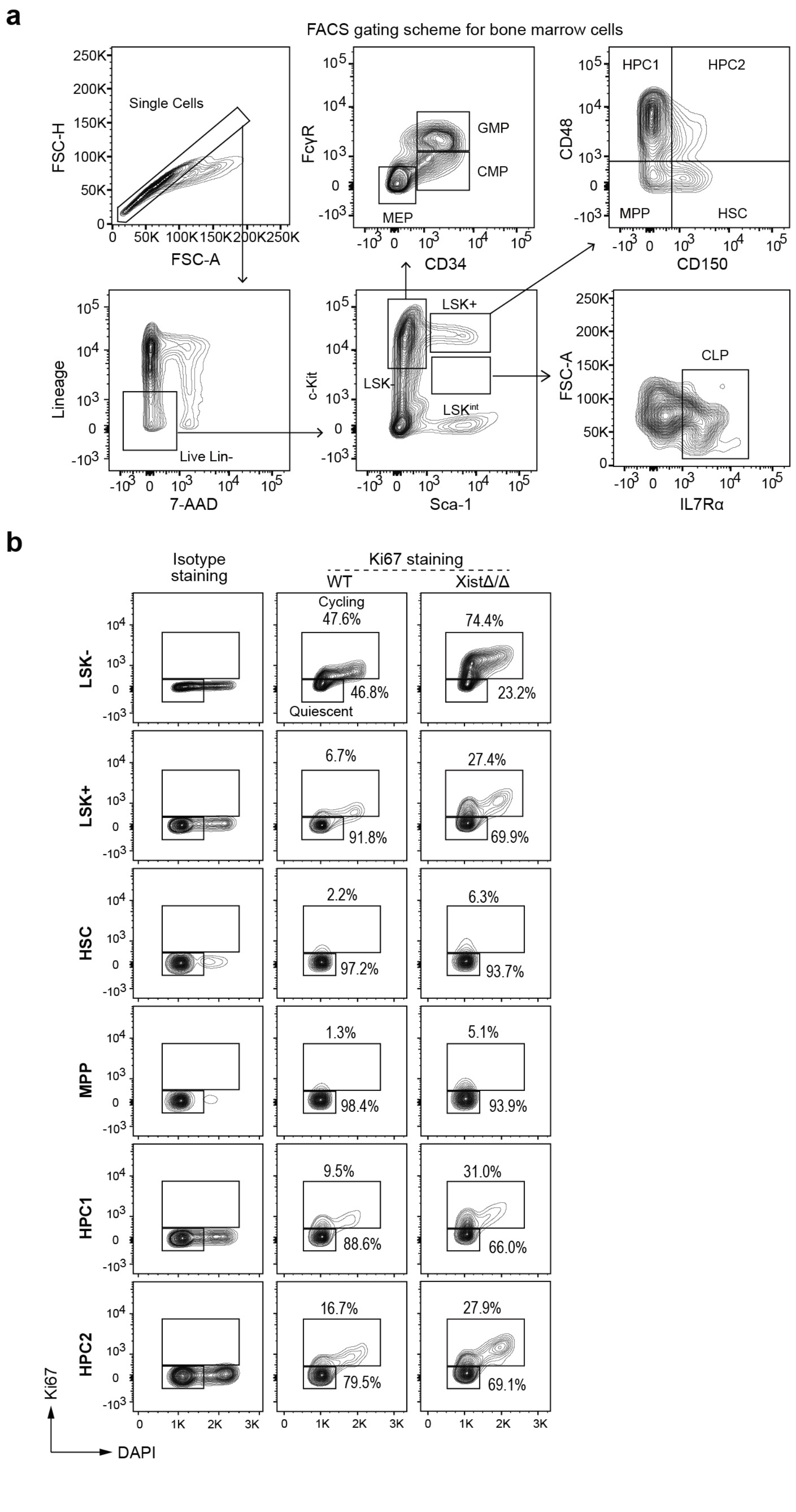


**Supplementary Fig. 1: FACS gating schemes for identification and cell cycle analysis of HSPC populations. a** Representative FACS gating scheme for identification of LSK+ cells,

LSK- cells, LSK+ subpopulations (HSC, MPP, HPC1/2), LSK- subpopulations (CMP, GMP and MEP) and lymphoid progenitors (CLP) in the mouse bone marrow. **b** Representative FACS gating scheme for Ki67 cell cycle analysis of LSK- cells, LSK+ cells and LSK+ cell subpopulations from a pair of 3-month-old WT and XistΔ/Δ female mice.

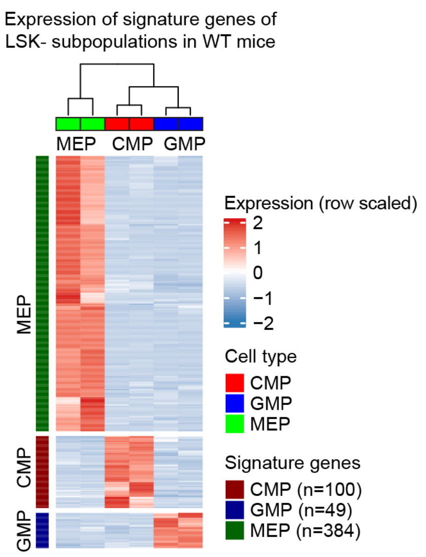


**Supplementary Fig. 2: Expression of signature genes of LSK- cell subpopulations.** Heatmap shows gene expression of LSK- subpopulation (CMP, GMP and MEP) signature genes in WT mice.

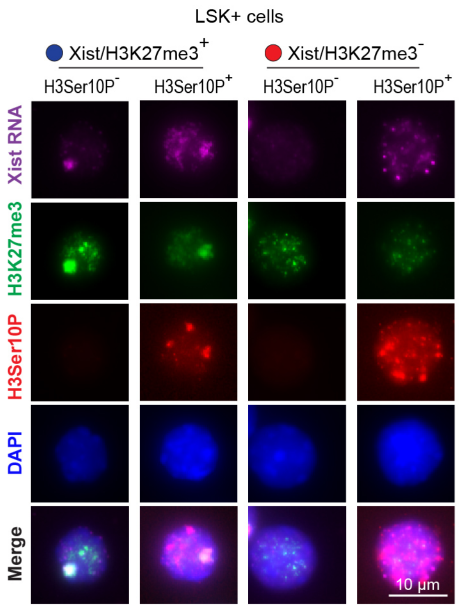


**Supplementary Fig. 3: Representative images of sequential Xist RNA FISH and IF staining of H3K27me3 and H3Ser10P in LSK+ cells directly isolated from the mouse bone marrow.** See Fig. 2j and 2k for the quantification of the number and percentage of Xist/H3K27me3$^{+}$ and Xist/H3K27me3$^{-}$ cells with or without H3Ser10P signal. LSK+ cells were isolated from several WT or Xist mutant female mice at 3- or 5-months old of age (n ≥3 per genotype and per age) and pooled for each genotype and age during staining assays and analysis.

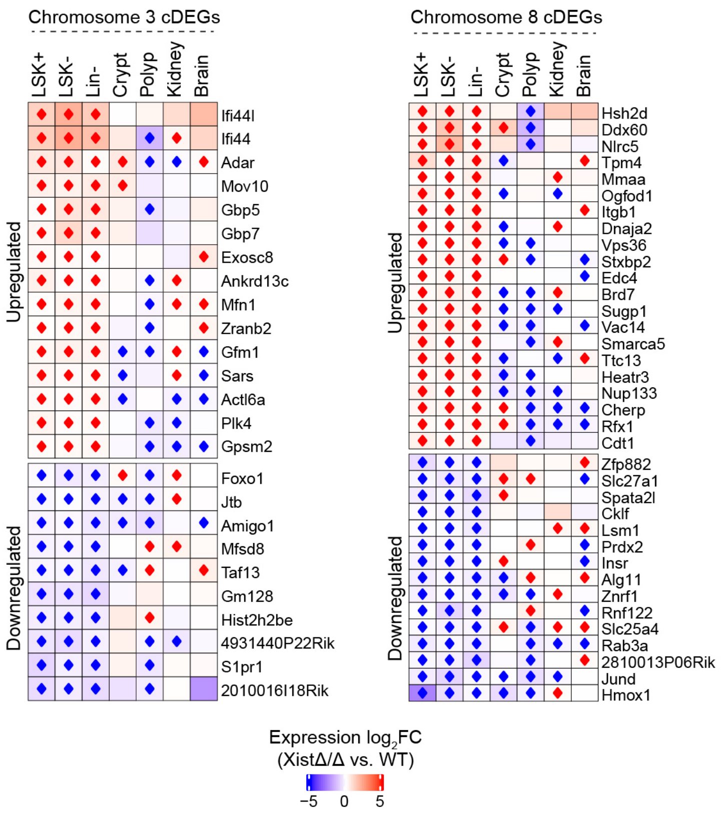


**Supplementary Fig. 4: Differential transcriptional changes in chromosome 3- and 8-specific cDEGs in HSPCs compared to various tissues.** Heatmaps show expression $\text{Log}_2\text{FC}$ of chromosome 3- and 8-linked cDEG in XistΔ/Δ and WT bone marrow cells (LSK+, LSK- and Lin- cells) in comparison to various tissues including brain [1], gut (crypt, polyp) and kidney [2]. Genes that are transcriptionally upregulated (red) or downregulated (blue) in both biological replicates (DEGs) are marked by rhombuses.

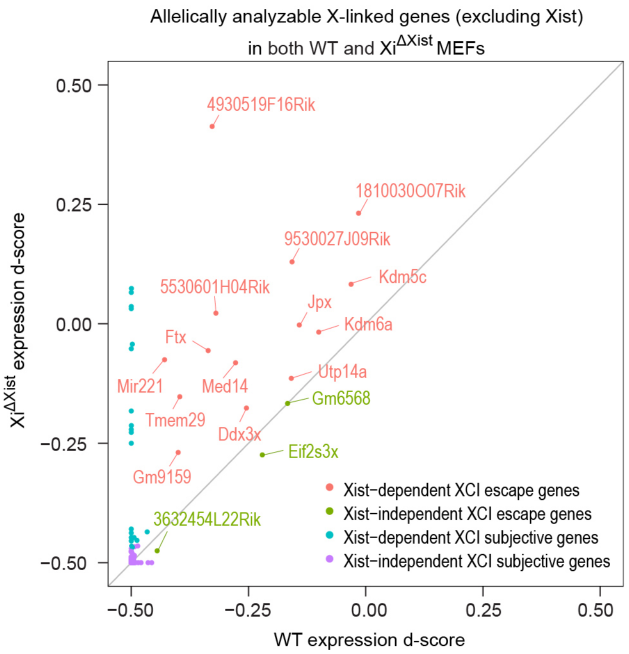


**Supplementary Fig. 5: Allele-specific analysis of X-linked gene expression in MEFs.** Scatter plot shows expression d-scores of different groups of allelically analyzable X-linked genes (excluding Xist) in WT and Xi$^{\Delta Xist}$ MEFs. Names of XCI escape genes are indicated.

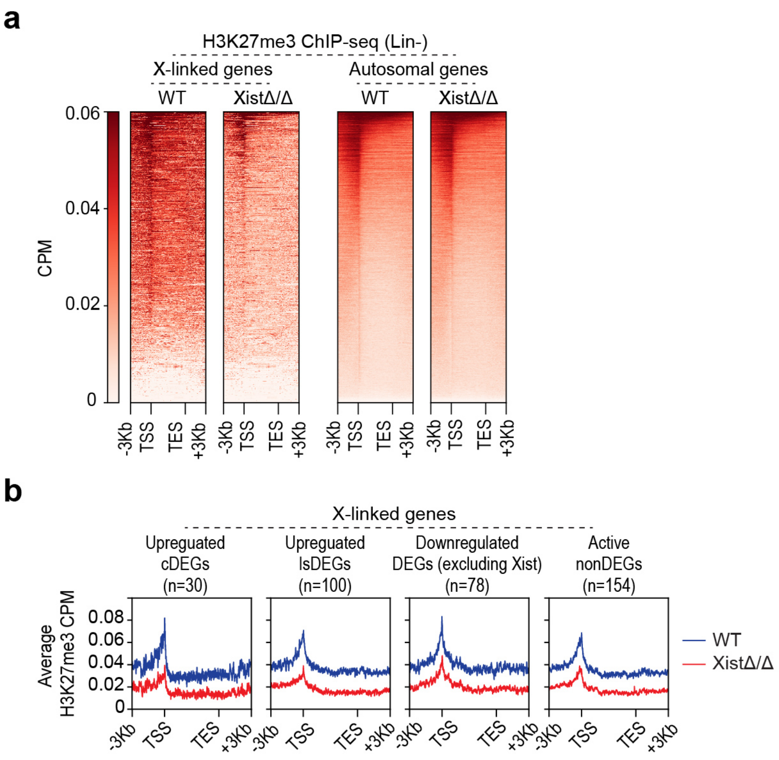


**Supplementary Fig. 6: Analysis of H3K27me3 distribution by ChIP-seq in Lin- HSPCs. a** Heat maps show H3K27me3 ChIP-seq signal across all X-linked and autosomal genes in Lin- cells isolated from 3-month-old WT and XistΔ/Δ female mice. CPM, counts per million reads. **b** Metagene profiles show distribution of average H3K27me3 ChIP-seq signal of upregulated cDEGs, upregulated lsDEGs, downregulated DEGs and active nonDEGs on X chromosome in Lin- cells. Number of genes in each group are as indicated. TSS, transcription start site; TES, transcription end site.

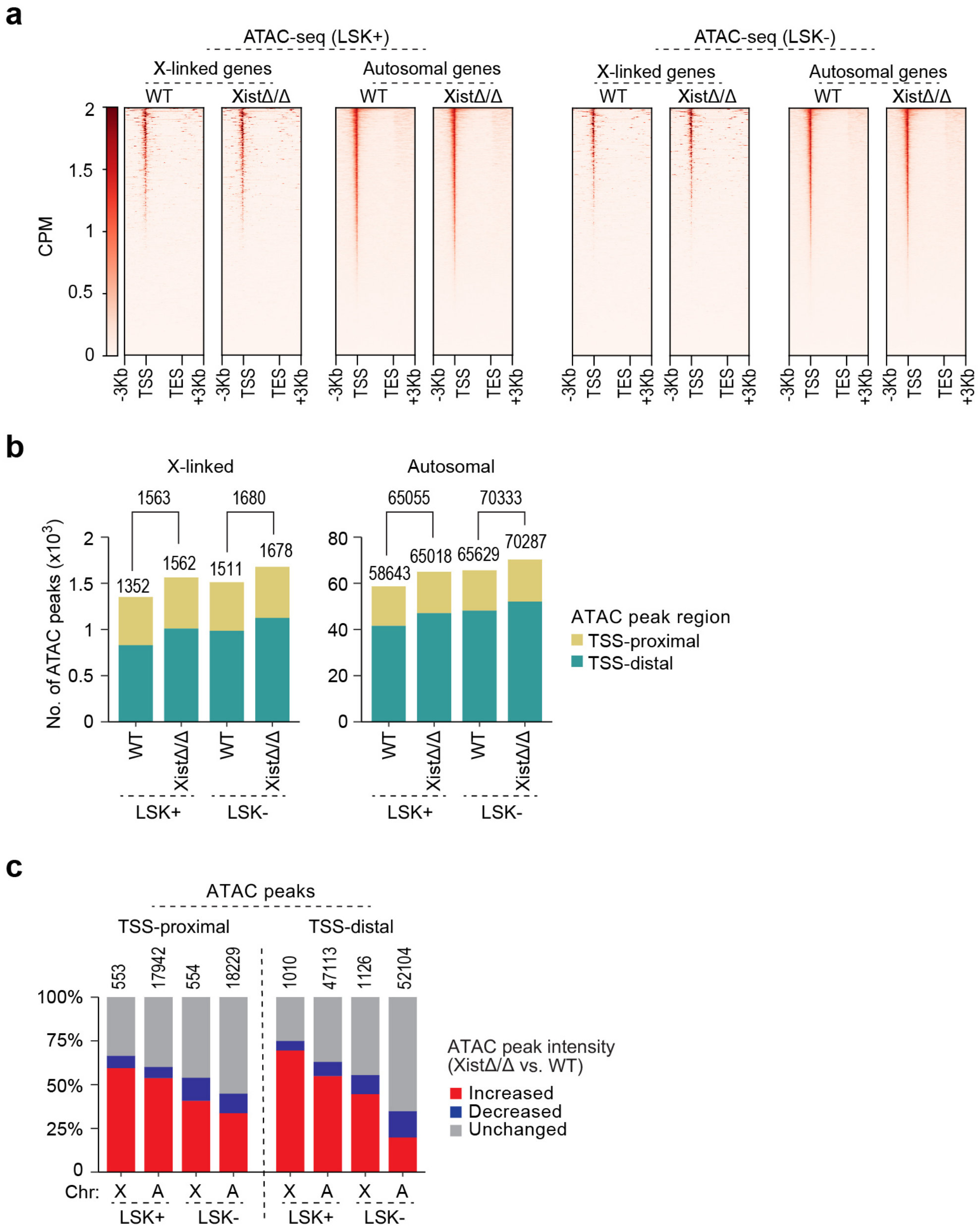


**Supplementary Fig. 7: Analysis of chromatin accessibility by ATAC-seq in LSK+ and LSK- cells. a** Heat maps show ATAC-seq signal across all X-linked and autosomal genes in LSK+ and LSK- cells isolated from 4.5-month-old WT and XistΔ/Δ female mice. CPM, counts per million. **b** Bar graphs show the number of ATAC peaks located at TSS-proximal and TSS-distal regions on the X chromosome and autosomes in LSK+ and LSK- cells from WT and XistΔ/Δ female mice. **c** Bar graphs show the percentages of increased, decreased, and unchanged ATAC peaks located at TSS-proximal and TSS-distal regions on the X chromosome and autosomes in LSK+ and LSK- cells. ATAC peak numbers are as indicated (**b**, **c**). TSS, transcription start site; TES, transcription end site.

**a**

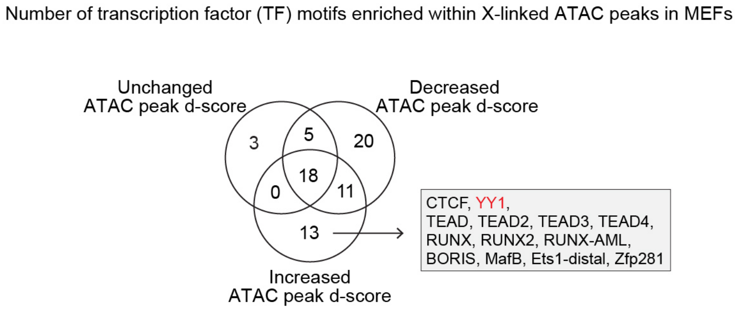


**b**

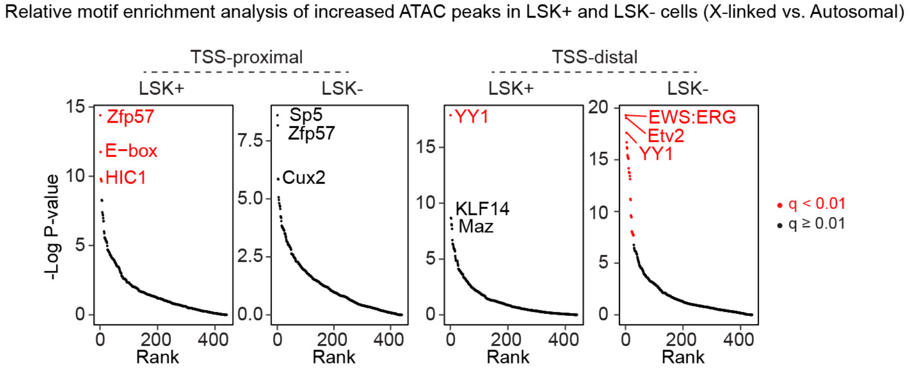


**Supplementary Fig. 8: Motif enrichment analysis of differential ATAC peaks in MEFs and HSPCs. a** Venn diagram shows the number of transcription factor (TF) binding motifs that are significantly enriched ($p < 0.01$ and $q < 0.01$) in increased, decreased, and unchanged X-linked ATAC peaks in MEFs. Motifs that are uniquely enriched in allelically increased ATAC peaks are indicated. See Supplementary Data 6 for complete results of motif enrichment analysis. **b** Relative motif enrichment analysis of X-linked increased ATAC peaks in comparison to autosomal increased ATAC peaks at TSS-proximal and TSS-distal regions in LSK+ and LSK- cells. Significantly enriched ($p < 0.01$ and $q < 0.01$) motifs are highlighted in red. The names of top 3 enriched motifs are indicated in each plot. See Supplementary Data 3 for complete results of relative motif enrichment analysis. Enrichment p-values were calculated by hypergeometric test and q-values were obtained by Benjamini-Hochberg adjustment according to default algorithm for HOMER motif analysis.

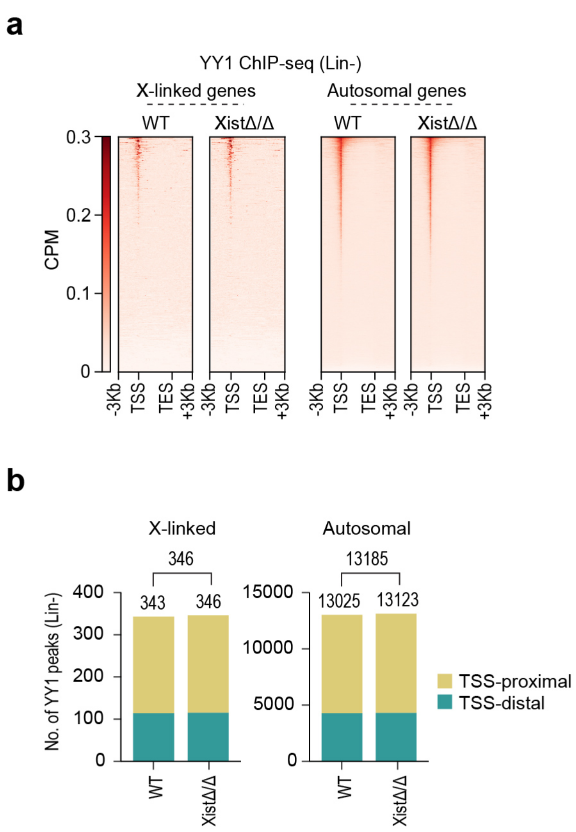


**Supplementary Fig. 9: Analysis of YY1 binding by ChIP-seq in Lin- HSPCs. a** Heat maps of YY1 ChIP-seq signal across all X-linked and autosomal genes in WT and XistΔ/Δ Lin- cells. CPM, counts per million. **b** Bar graphs show the number of YY1 peaks located at TSS-proximal and TSS-distal regions on the X chromosome and autosomes in WT and XistΔ/Δ Lin- cells. YY1 peak numbers are as indicated. TSS, transcription start site; TES, transcription end site.

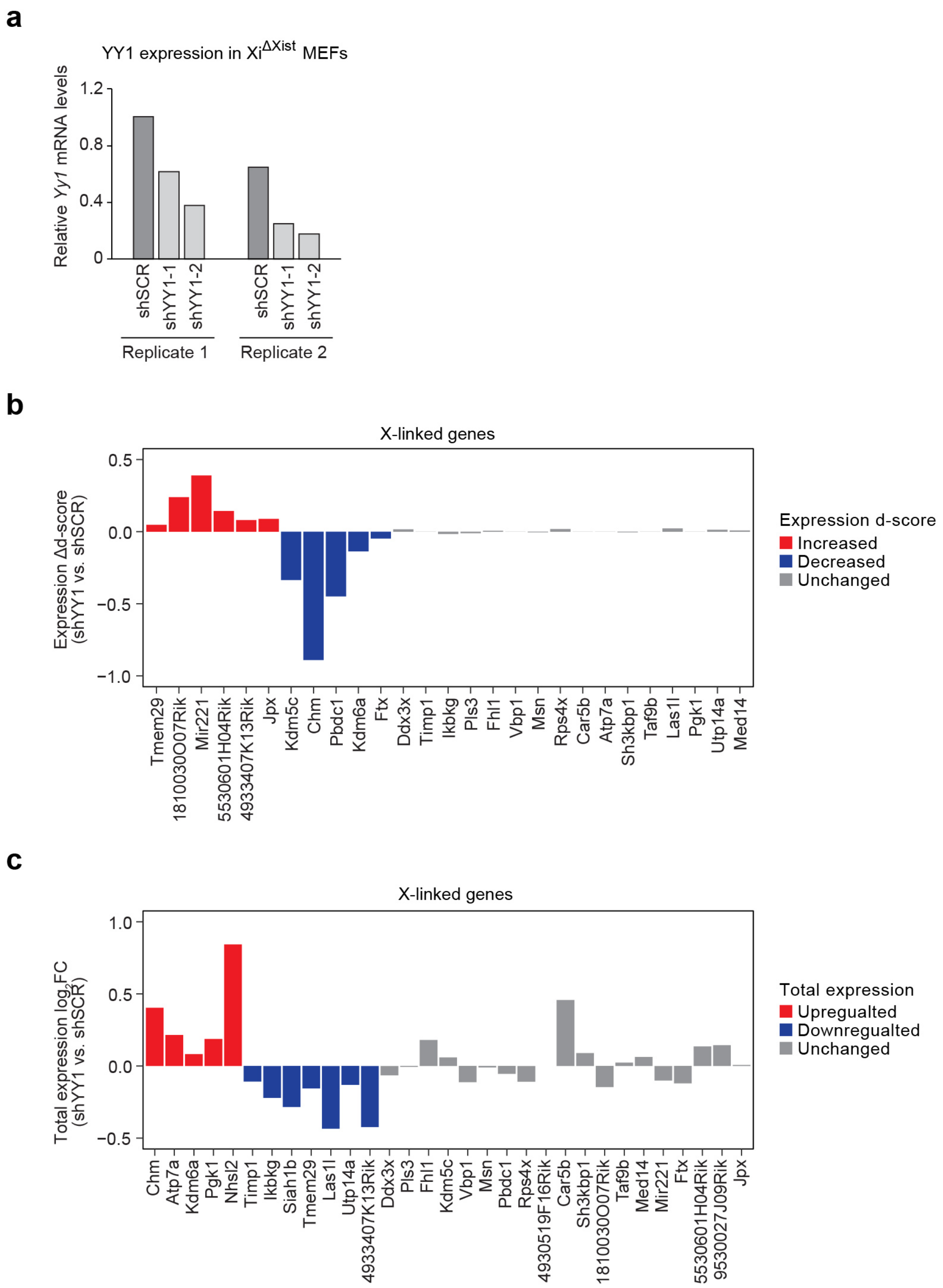


**Supplementary Fig. 10: Knockdown of YY1 expression in MEFs by shRNA. a** Real time RT-PCR analysis of YY1 expression in control (shSCR) and YY1 shRNA (shYY1-1, shYY1-2) lentivirus transduced Xi$^{\Delta Xist}$ MEFs. *Gapdh* mRNA level was used to normalize mRNA levels. **b-c** Bar graphs show changes in expression d-score (**b**) and total expression (**c**) of Xist-dependent genes between shSCR and shYY1 transduced Xi$^{\Delta Xist}$ MEFs. Source data are provided as a Source Data file.

**a**

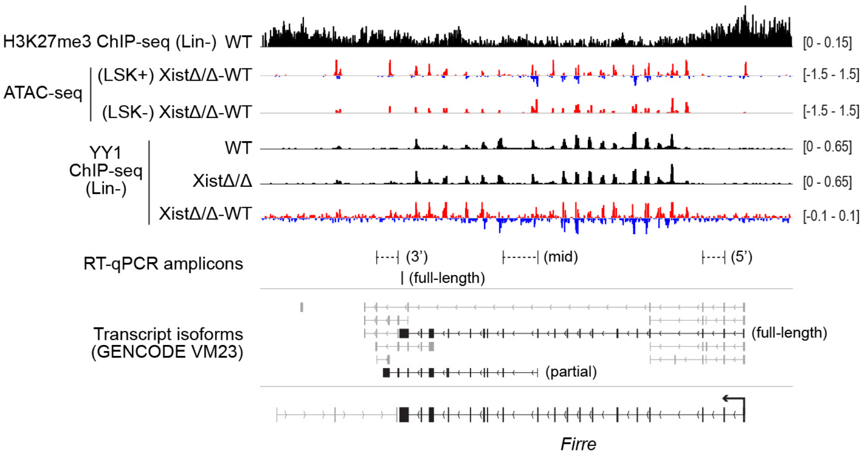


**b**

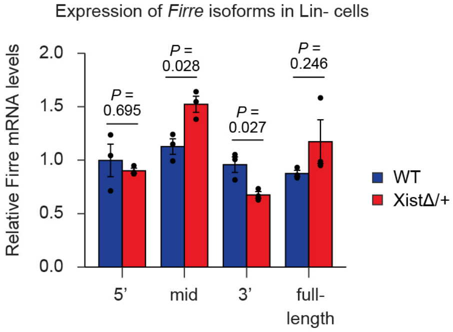


**Supplementary Fig. 11: Analysis of YY1 binding and transcription at the *Firre* locus in HSPCs. a** Representative genome tracks show distribution of YY1, H3K27me3, H3K27ac, and ATAC signals across *Firre* locus in Lin- cells. Splice variants of Firre gene and real time RT-PCR amplicon positions within the *Firre* locus are as shown. **b** Real time RT-PCR showing relative *Firre* isoform mRNA levels in XistΔ/+ and WT bone marrow Lin- cells. *Gapdh* was used to normalize mRNA levels. The partial *Firre* transcript isoform (mid) is upregulated in Xist-deficient cells. All error bars represent standard error of the mean (SEM) and data are presented as mean values +/- SEM. P-values were calculated using two-tailed unpaired Student's *t*-test. Source data are provided as a Source Data file.

## SUPPLEMENTARY REFERENCES


1. Adrianse, R.L. *et al.* Perturbed maintenance of transcriptional repression on the inactive X-chromosome in the mouse brain after Xist deletion. *Epigenetics Chromatin* **11**, 50 (2018).
2. Yang, L., Yildirim, E., Kirby, J.E., Press, W. & Lee, J.T. Widespread organ tolerance to Xist loss and X reactivation except under chronic stress in the gut. *Proc Natl Acad Sci U S A* **117**, 4262-4272 (2020).
