# Supplementary material for: Xist exerts gene-specific silencing during XCI maintenance and impacts lineage-specific cell differentiation and proliferation during hematopoiesis
Source: Nat Commun. 2022 Aug 1;13:4464. doi: 10.1038/s41467-022-32273-5 (PMC9343370; doi:10.1038/s41467-022-32273-5)
Supplement: Supplementary file 2 — Description of Additional Supplementary Files [file 41467_2022_32273_MOESM2_ESM.pdf]

Description of Additional Supplementary Files

Xist exerts gene-specific silencing during XCI maintenance and impacts lineage-specific cell differentiation and proliferation during hematopoiesis

Yang *et al.*

Supplementary Data 1: Transcriptional analysis of WT and Xist-deficient LSK+, LSK- and Lin- cells.
Related to Fig. 3a-e and Supplementary Fig. 4.

Supplementary Data 2: H3K4me3 and H3K27me3 ChIP-seq analysis of WT and Xist-deficient Lin- cells.
Related to Fig. 3f, 4b.

Supplementary Data 3: ATAC-seq analysis of WT and Xist-deficient LSK+ and LSK- cells.
Related to Fig. 3f, 4c and Supplementary Fig. 7b-c, 8b.

Supplementary Data 4: Allele-specific transcriptional analysis of WT and Xist-deficient MEFs.
Related to Fig. 3h and Supplementary Fig. 5.

Supplementary Data 5: Allele-specific H3K27me3 ChIP-seq analysis of WT and Xist-deficient MEFs.
Related to Fig. 5a.

Supplementary Data 6: Allele-specific analysis of previously published ATAC-seq data of WT and Xist-deficient MEFs.
Related to Fig. 5b and Supplementary Fig. 8a.

Supplementary Data 7: Allele-specific H3K27ac ChIP-seq analysis of WT and Xist-deficient MEFs.
Related to Figure 5a-d, 6b.

Supplementary Data 8: YY1 ChIP-seq analysis of WT and Xist-deficient Lin- cells.
Related to Fig. 6a, 6d and Supplementary Fig. 9b.

Supplementary Data 9: Allele-specific YY1 ChIP-seq analysis of WT and Xist-deficient MEFs.
Related to Fig. 6b, 6e and 6h.

Supplementary Data 10: Allele-specific transcriptional analysis of Xist-dependent genes in Xist-deficient MEFs upon YY1 knockdown by shRNA.
Related to Supplementary Fig. 10.

Supplementary Data 11: List containing name, genotype, platform, sequencing type, and depth for all RNA-, ATAC-, and ChIP-seq samples and data.
