# Supplementary material for: Xist exerts gene-specific silencing during XCI maintenance and impacts lineage-specific cell differentiation and proliferation during hematopoiesis
Source: Nat Commun. 2022 Aug 1;13:4464. doi: 10.1038/s41467-022-32273-5 (PMC9343370; doi:10.1038/s41467-022-32273-5)
Supplement: Supplementary file 14 — Reporting Summary [file 41467_2022_32273_MOESM14_ESM.pdf]

nature portfolio

Corresponding author(s): Eda Yildirim

Last updated by author(s): Jul 15, 2022

Reporting Summary

Nature Portfolio wishes to improve the reproducibility of the work that we publish. This form provides structure for consistency and transparency in reporting. For further information on Nature Portfolio policies, see our Editorial Policies and the Editorial Policy Checklist.

Statistics

For all statistical analyses, confirm that the following items are present in the figure legend, table legend, main text, or Methods section.

n/a	Confirmed	
	☒	The exact sample size (*n*) for each experimental group/condition, given as a discrete number and unit of measurement
	☒	A statement on whether measurements were taken from distinct samples or whether the same sample was measured repeatedly
	☒	The statistical test(s) used AND whether they are one- or two-sided<br>*Only common tests should be described solely by name; describe more complex techniques in the Methods section.*
☒		A description of all covariates tested
☒		A description of any assumptions or corrections, such as tests of normality and adjustment for multiple comparisons
☒		A full description of the statistical parameters including central tendency (e.g. means) or other basic estimates (e.g. regression coefficient) AND variation (e.g. standard deviation) or associated estimates of uncertainty (e.g. confidence intervals)
	☒	For null hypothesis testing, the test statistic (e.g. *F*, *t*, *r*) with confidence intervals, effect sizes, degrees of freedom and *P* value noted<br>*Give P values as exact values whenever suitable.*
☒		For Bayesian analysis, information on the choice of priors and Markov chain Monte Carlo settings
☒		For hierarchical and complex designs, identification of the appropriate level for tests and full reporting of outcomes
	☒	Estimates of effect sizes (e.g. Cohen's *d*, Pearson's *r*), indicating how they were calculated

Our web collection on statistics for biologists contains articles on many of the points above.

Software and code

Policy information about availability of computer code

Data collection	Flourescent in situ hybridization and immunoflourescence images were collected with a Leica DM5500B microscope and Leica DFC365 FX CCD camera.
Data analysis	Software used has been described in "Methods". Please also find a list below:<br>bedtools (v2.25.0)<br>Bowtie2 (v2.3.5.1)<br>ChIPpeakAnno R package (v3.24.1)<br>clusterProfiler R package (v3.18.1)<br>deepTools (v3.5.0)<br>DEseq2 (v1.30.1)<br>DiffBind R package (v3.0.13)<br>featureCounts (v1.6.3)<br>FastQC (0.11.7)<br>Fiji (v2.3.0/1.53f)<br>FlowJo (v10.8.1)<br>GSEA software (v4.0.3)<br>HOMER (v4.11)<br>MACS2 (v2.2.7.1)<br>Picard (v2.0.1)<br>Samtools (v1.10)<br>SNPsplit (v0.3.2)<br>TopHat (v2.1.1)

TrimGalore (v0.6.0)

The custom analysis pipelines for all genomic analyses are available upon request with no restrictions.

For manuscripts utilizing custom algorithms or software that are central to the research but not yet described in published literature, software must be made available to editors and reviewers. We strongly encourage code deposition in a community repository (e.g. GitHub). See the Nature Portfolio guidelines for submitting code & software for further information.

Data

Policy information about availability of data

All manuscripts must include a data availability statement. This statement should provide the following information, where applicable:

- Accession codes, unique identifiers, or web links for publicly available datasets
- A description of any restrictions on data availability
- For clinical datasets or third party data, please ensure that the statement adheres to our policy

All NGS data generated in this study have been uploaded to the Gene Expression Omnibus (GEO), under GSE184776 (https://www.ncbi.nlm.nih.gov/geo/query/acc.cgi?acc=GSE184776).
Accession number and hyperlinks for previously published and publicly available sequencing data that were reanalyzed in this study are listed below:
E-MTAB-8161 (https://www.ebi.ac.uk/arrayexpress/experiments/E-MTAB-8161/)
GSE97077 (https://www.ncbi.nlm.nih.gov/geo/query/acc.cgi?acc=GSE97077)
GSE109395 (https://www.ncbi.nlm.nih.gov/geo/query/acc.cgi?acc=GSE109395)

Field-specific reporting

Please select the one below that is the best fit for your research. If you are not sure, read the appropriate sections before making your selection.

[x] Life sciences [ ] Behavioural & social sciences [ ] Ecological, evolutionary & environmental sciences

For a reference copy of the document with all sections, see nature.com/documents/nr-reporting-summary-flat.pdf

Life sciences study design

All studies must disclose on these points even when the disclosure is negative.

Sample size	Not relevant for this study. No human or animal subjects necessitating sample size calculations were used in this study
Data exclusions	No data were excluded from analysis or reporting.
Replication	The number of biological replicates of RNA-, ChIP- and ATAC- seq experiments were noted in Supplementary information. At least 2 biological replicates of sequencing experiments were conducted for all seq experiments except for H3K27me3 ChIP-seq in MEFs. At least 3 biological replicates of FACS experiments were conducted. No replicates were excluded from analyses presented, and all attempts at replication were successful. The number of cells and biological replicates for immuno-FISH experiments were noted in related Figures and Methods section.
Randomization	No such relevant experiments.
Blinding	No requirement for the blinding test.

Reporting for specific materials, systems and methods

We require information from authors about some types of materials, experimental systems and methods used in many studies. Here, indicate whether each material, system or method listed is relevant to your study. If you are not sure if a list item applies to your research, read the appropriate section before selecting a response.

Materials & experimental systems

n/a	Involved in the study	
[ ]	[x]	Antibodies
[ ]	[x]	Eukaryotic cell lines
[x]	[ ]	Palaeontology and archaeology
[ ]	[x]	Animals and other organisms
[x]	[ ]	Human research participants
[x]	[ ]	Clinical data
[x]	[ ]	Dual use research of concern

Methods

n/a	Involved in the study	
[ ]	[x]	ChIP-seq
[ ]	[x]	Flow cytometry
[x]	[ ]	MRI-based neuroimaging

Antibodies

Antibodies used

FACS antibodies:
BV421-conjugated Streptavidin BD 563259
FITC-conjugated anti-Ly6A/E (Sca1) BD 557405
PE-conjugated anti-CD117 (c-Kit) BD 553355
APC/Cy7-conjugated anti-CD48 Biolegend 103432
PE/Cy7-conjugated anti-CD150 Biolegend 115914
PE/Cy7-conjugated anti-CD16/32 (FcγRIII/II) BD 560829
PE/Cy7-conjugated anti-CD127 (IL7Rα) BD 560733
PerCP-conjugated Streptavidin BD 554064
AlexaFluor647-conjugated anti-CD34 BD 560230 Clone: RAM34
AlexaFluor647-conjugated anti-Ki67 Biolegend 652408 Clone: 16A8
AlexaFluor674-conjugated anti-IgG2 antibody BioLegend 400526 Clone: RTK2758
Biotin-conjugated anti-CD11b (MacI) BD 553309
Biotin-conjugated anti-CD3ε BD 553060
Biotin-conjugated anti-CD4 BD 553728
Biotin-conjugated anti-CD45R (B220) BD 553086
Biotin-conjugated anti-CD8α BD 553029
Biotin-conjugated anti-Ly6G (Gr-1) BD 553125
Biotin-conjugated anti-Ter119 BD 553672

FISH and IF antibodies:
AlexaFluor647-conjugated Streptavidin Invitrogen S21374
anti-H3K27me2/me3 Active Motif 39535 Clone: 7B11
anti-H3K27me3 Cell Signaling 9733 Clone: C36B11
anti-H3Ser10P Millipore 05-806 Clone: 3H10
AlexaFluor488-conjugated goat anti-rabbit IgG (H&L) ThermoFisher A-11008
AlexaFluor555-conjugated goat anti-mouse IgG (H&L) ThermoFisher A-21422
AlexaFluor555-conjugated goat anti-rabbit IgG antibody (H&L) Invitrogen A32732

ChIP antibodies:
anti-H3K27me3 Abcam ab6002 Clone: mAbcam6002
anti-H3K27ac antibody Abcam ab4729
anti-H3K4me3 Abcam ab8580
anti-YY1 Cell Signaling 46395

Validation

All FACS antibodies listed above were validated by flow cytometry using mouse bone marrow cells. All ChIP antibodies were validated by western blotting using MEF cells. Notably, all antibodies used for FACS, ChIP or IF experiments are well established clones and every lot is quality tested by the supplier using immunofluorescence staining or using specific techniques including ChIP or IF. Each antibody was titrated by us for optimal performance and optimal concentrations are indicated under Methods. Citations including the validation of antibodies are visible on the manufacturer websites. See Methods section and above for details on the manufacturer name and catalog number for each antibody.

Eukaryotic cell lines

Policy information about cell lines

Cell line source(s)

WT and XaWT XiΔXist fibroblast lines (Zhang et al., 2007 Cell).
HEK293T cells were obtained from the American Tissue Collection Center (ATCC, Manassas, VA, USA) through the Duke University Cancer Center Facilities.

Authentication

Fibroblast lines were genotyped by PCR (Zhang et al., 2007 Cell). HEK293T cells were authenticated by the Duke University DNA Analysis Facility. The Facility uses the GenePrint 10 kit (Promega). Using this kit, polymorphic short tandem repeat (STR) markers were analyzed.

Mycoplasma contamination

All cell lines used in the study tested negative for mycoplasma.

Commonly misidentified lines
(See ICLAC register)

No commonly misidentified lines were used.

Animals and other organisms

Policy information about studies involving animals; ARRIVE guidelines recommended for reporting animal research

Laboratory animals

Xist2lox/2lox mice (129Sv4/Jae strain) and B6.Cg-Commd10Tg (Vav1-cre) A2Kio/J (Vav-iCre, Jax 008610) mice were obtained from the Mutant Mouse Resource and Research Center (MMRRC) at the University of North Carolina (UNC) and The Jackson Laboratory, respectively. X chromosome inactivation only occurs in the female organism. In this study, we investigated the role of Xist RNA in hematopoiesis and utilized only WT and XistΔ/Δ female mice throughout the study. Age of mice ranged from 3 to 5 months old.

	Number of mice utilized are indicated in related figures and/or under the Methods section. Mouse husbandry and experiments were all carried out as stipulated by the Duke University Institutional Animal Care and Use Committee (IACUC). Mouse housing conditions were in a 12 h light-dark cycle at room temperature (20–23 °C) with 40–70% humidity with ad libitum access to food and filtered water. Environmental enrichment, such as mouse houses or and nesting material were provided to all animals.
Wild animals	No wild animals were used in the study.
Field-collected samples	No field-collected samples were used in the study.
Ethics oversight	Mouse husbandry and experiments were carried out as stipulated by the Duke University Institutional Animal Care and Use Committee (IACUC).

Note that full information on the approval of the study protocol must also be provided in the manuscript.

ChIP-seq

Data deposition

- [x] Confirm that both raw and final processed data have been deposited in a public database such as GEO.
- [x] Confirm that you have deposited or provided access to graph files (e.g. BED files) for the called peaks.

Data access links<br>*May remain private before publication.*	Raw and processed sequencing data have been deposited in the Gene Expression Omnibus (GEO) under accession number GSE184776 (https://www.ncbi.nlm.nih.gov/geo/query/acc.cgi?acc=GSE184776).
Files in database submission	ChIP-seq raw data files:<br>6793-L5_S4_L002_R1_001.fastq.gz<br>6793-L5_S4_L002_R2_001.fastq.gz<br>6793-L7_S6_L002_R1_001.fastq.gz<br>6793-L7_S6_L002_R2_001.fastq.gz<br>6867-L1_S26_L002_R1_001.fastq.gz<br>6867-L1_S26_L002_R2_001.fastq.gz<br>6867-L3_S28_L002_R1_001.fastq.gz<br>6867-L3_S28_L002_R2_001.fastq.gz<br>EY-S53_S8_L003_R1_001.fastq.gz<br>EY-S54_S9_L003_R1_001.fastq.gz<br>EY-S56_S11_L004_R1_001.fastq.gz<br>EY-S57_S12_L004_R1_001.fastq.gz<br>EY-S59_S14_L005_R1_001.fastq.gz<br>EY-S60_S15_L005_R1_001.fastq.gz<br>EY-S62_S17_L006_R1_001.fastq.gz<br>EY-S63_S18_L006_R1_001.fastq.gz<br>S03_L1_CAGTGCTT_V300044250_L1_R1.fq.gz<br>S03_L1_CAGTGCTT_V300044250_L1_R2.fq.gz<br>S03_L2_CAGTGCTT_V300044250_L2_R1.fq.gz<br>S03_L2_CAGTGCTT_V300044250_L2_R2.fq.gz<br>S04_L1_TCCATTGC_V300044250_L1_R1.fq.gz<br>S04_L1_TCCATTGC_V300044250_L1_R2.fq.gz<br>S04_L2_TCCATTGC_V300044250_L2_R1.fq.gz<br>S04_L2_TCCATTGC_V300044250_L2_R2.fq.gz<br>6793-L1_S1_L002_R1_001.fastq.gz<br>6793-L1_S1_L002_R2_001.fastq.gz<br>6793-L2_S1_L002_R1_001.fastq.gz<br>6793-L2_S1_L002_R2_001.fastq.gz<br>6793-L3_S2_L002_R1_001.fastq.gz<br>6793-L3_S2_L002_R2_001.fastq.gz<br>L1_CKDL220003402-1a_HGFJKDSX3_L1_1.fq.gz<br>L1_CKDL220003402-1a_HGFJKDSX3_L1_2.fq.gz<br>L2_CKDL220003403-1a_HGFJKDSX3_L1_1.fq.gz<br>L2_CKDL220003403-1a_HGFJKDSX3_L1_2.fq.gz<br>L3_CKDL220003404-1a_HGFJKDSX3_L1_1.fq.gz<br>L3_CKDL220003404-1a_HGFJKDSX3_L1_2.fq.gz<br>L4_CKDL220003405-1a_HGFJKDSX3_L1_1.fq.gz<br>L4_CKDL220003405-1a_HGFJKDSX3_L1_2.fq.gz<br>L5_CKDL220003406-1a_HGFJKDSX3_L1_1.fq.gz<br>L5_CKDL220003406-1a_HGFJKDSX3_L1_2.fq.gz<br>L6_CKDL220003407-1a_HGFJKDSX3_L1_1.fq.gz<br>L6_CKDL220003407-1a_HGFJKDSX3_L1_2.fq.gz<br>L7_CKDL220003408-1a_HGFJKDSX3_L1_1.fq.gz<br>L7_CKDL220003408-1a_HGFJKDSX3_L1_2.fq.gz

L8_CKDL220003409-1a_HGFJKDSX3_L1_1.fq.gz
L8_CKDL220003409-1a_HGFJKDSX3_L1_2.fq.gz

ChIP-seq processed data files:
Mouse_Lin_F_WT_H3K27me3_ChIPseq_SE_4601_rep1.CPM.bigwig
Mouse_Lin_F_WT_H3K27me3_ChIPseq_SE_4601_rep2.CPM.bigwig
Mouse_Lin_F_Xist.dKO_H3K27me3_ChIPseq_SE_4601_rep1.CPM.bigwig
Mouse_Lin_F_Xist.dKO_H3K27me3_ChIPseq_SE_4601_rep2.CPM.bigwig
Mouse_Lin_F_WT_H3K4me3_ChIPseq_SE_4601_rep1.CPM.bigwig
Mouse_Lin_F_WT_H3K4me3_ChIPseq_SE_4601_rep2.CPM.bigwig
Mouse_Lin_F_Xist.dKO_H3K4me3_ChIPseq_SE_4601_rep1.CPM.bigwig
Mouse_Lin_F_Xist.dKO_H3K4me3_ChIPseq_SE_4601_rep2.CPM.bigwig
Mouse_Lin_F_WT_YY1_ChIPseq_PE_6793_rep1_homerpeaks.bed
Mouse_Lin_F_WT_YY1_ChIPseq_PE_6867_rep2_homerpeaks.bed
Mouse_Lin_F_Xist.dKO_YY1_ChIPseq_PE_6793_rep1_homerpeaks.bed
Mouse_Lin_F_Xist.dKO_YY1_ChIPseq_PE_6867_rep2_homerpeaks.bed
MEF_WT_H3K27ac_129.bigwig
MEF_WT_H3K27ac_CAST.bigwig
MEF_WT_H3K27me3_129.bigwig
MEF_WT_H3K27me3_CAST.bigwig
MEF_WT_YY1_129.bigwig
MEF_WT_YY1_CAST.bigwig
MEF_XistKO_H3K27ac_129.bigwig
MEF_XistKO_H3K27ac_CAST.bigwig
MEF_XistKO_H3K27me3_129.bigwig
MEF_XistKO_H3K27me3_CAST.bigwig
MEF_XistKO_YY1_129.bigwig
MEF_XistKO_YY1_CAST.bigwig

Genome browser session (e.g. UCSC)

Not applicable.

Methodology

Replicates

The number of biological replicates of ChIP-seq experiments were noted in Supplementary information. At least 2 biological replicates of sequencing experiments were conducted for all seq experiments except for H3K27me3 ChIP-seq in MEFs.

Sequencing depth

Lin- H3K4me3 ChIP-seq (WT rep1) SE100 111167277
Lin- H3K4me3 ChIP-seq (WT rep2) SE100 98847627
Lin- H3K27me3 ChIP-seq (WT rep1) SE100 112521757
Lin- H3K27me3 ChIP-seq (WT rep2) SE100 117468371
Lin- H3K27me3 ChIP-seq (XistΔ/Δ rep1) SE100 111377040
Lin- H3K27me3 ChIP-seq (XistΔ/Δ rep2) SE100 95950290
Lin- YY1 ChIP-seq (WT rep1) PE50 105253958
Lin- YY1 ChIP-seq (WT rep2) PE50 198724572
Lin- YY1 ChIP-seq (XistΔ/Δ rep1) PE50 78965280
Lin- YY1 ChIP-seq (XistΔ/Δ rep2) PE50 197004666
MEF H3K27me3 ChIP-seq (WT) PE100 295942134
MEF H3K27me3 ChIP-seq (XiΔXist) PE100 165523392
MEF YY1 ChIP-seq (WT rep1) PE150 58392586
MEF YY1 ChIP-seq (WT rep2) PE150 70807670
MEF YY1 ChIP-seq (WT rep3) PE50 88768268
MEF YY1 ChIP-seq (WT rep4) PE50 73557422
MEF YY1 ChIP-seq (XiΔXist rep1) PE150 62572062
MEF YY1 ChIP-seq (XiΔXist rep2) PE150 58585518
MEF YY1 ChIP-seq (XiΔXist rep3) PE50 98107712
MEF H3K27ac ChIP-seq (WT rep1) PE150 82732130
MEF H3K27ac ChIP-seq (WT rep2) PE150 68151418
MEF H3K27ac ChIP-seq (XiΔXist rep1) PE150 61819338
MEF H3K27ac ChIP-seq (XiΔXist rep2) PE150 98761222

Antibodies

The following antibodies were utilized for ChIP-seq experiments:
anti-H3K4me3; Abcam ab8580; ChIP grade
anti-H3K27me3; Abcam ab6002; ChIP grade; mAbcam 6002
anti-YY1; Cell Signaling 46395; Clone D5D9Z
anti-H3K27ac; Abcam, ab4729; ChIP grade

Peak calling parameters

For YY1 and H3K27ac ChIP-seq, peaks were called using findPeaks (-region -size 500 -minDist 500 -F 0 -L 4 -C 2) program in HOMER (v4.11).

Data quality	All fastq files passed per base sequence quality check by FastQC (v 0.11.5). The mapped reads were filtered by MAPQ greater than 30 by Samtools (v1.10).
Software	Bowtie2 (v2.3.5.1)<br>ChIPpeakAnno R package (v3.24.1)<br>FastQC (0.11.7)<br>MACS2 (v2.2.7.1)<br>Picard (v2.0.1)<br>Samtools (v1.10, -q 30)<br>SNPsplit (v0.3.2)<br>TrimGalore (v0.6.0)

Flow Cytometry

Plots

Confirm that:

- [x] The axis labels state the marker and fluorochrome used (e.g. CD4-FITC).
- [x] The axis scales are clearly visible. Include numbers along axes only for bottom left plot of group (a 'group' is an analysis of identical markers).
- [x] All plots are contour plots with outliers or pseudocolor plots.
- [x] A numerical value for number of cells or percentage (with statistics) is provided.

Methodology

Sample preparation	Mouse bones (tibias and femurs) were dissected, and bone marrow (BM) was flushed with 1xPBS supplemented with 2% FBS (2% F/PBS) into a 50-ml tube (Falcon) using a 22.5 gauge needle. Cell suspension was pipetted up and down for several times and filtered through a 70µm cell strainer (Falcon, 352350) to obtain a single-cell suspension. Red blood cells in the cell suspension were lysed by ACK Lysing buffer (Gibco, A10492-01) according to the manufacturer's instructions. To label lineage-positive cells, the following antibody cocktail containing biotinylated antibodies against mouse CD11b (BD, 553309, M1/70 clone; 1:200), CD3e (BD, 553060, 145-2C11 clone; 1:200), CD45R/B220 (BD, 553088, RA3-6B2 clone; 1:200), CD8a (BD, 553029, 53-6.7 clone; 1:200), CD4 (BD, 553728, GK1.5 clone; 1:200), Ly-6C/G (BD, 553125, RB6-8C5 clone; 1:200) and TER-119 (BD, 553672; 1:200) in combination with BV421-conjugated Streptavidin (BD, 563259; 1:200) was used. To distinguish between LSK+ and LSK- cells, biotinylated lineage-specific antibody cocktail was used in combination with FITC-conjugated Ly-6A/E (Sca-1) (BD, 557405, D7 clone; 1:100) and PE-conjugated CD117 (c-Kit) (BioLegend, 105808, 2B8 clone; 1:100) antibodies. To distinguish LSK+ subpopulations (HSC, MPP HPC1, HPC2), cells were further labelled with PE/Cy7-conjugated CD150 (BioLegend, 115914, TC15-12F12.2 clone; 1:100) and APC/Cy7-conjugated CD48 (BioLegend, 103432, HM48-1 clone; 1:100) antibodies. To distinguish LSK- subpopulations (MEP, GMP, CMP), cells were labelled with AlexaFluor647-conjugated CD34 (BD, 560230, RAM34 clone; 1:100) and PE/Cy7-conjugated CD16/32 (FcγR) (BD, 560829, 2.4G2 clone; 1:100) antibodies. To identify CLP, we stained cells with PE/Cy7-conjugated CD127 (IL7R)(BD, 560733, SB/199 clone; 1:100). 7-Amino-Actinomycin D (7-AAD) (BD, 559925; 1:200) solution was used to label the nonviable cells. Labelled cells were filtered through a 70µm cell strainer before FACS analysis or sorting.<br><br>For Ki67 cell cycle assay, bone marrow cells were labelled using biotinylated lineage antibody cocktail in combination with PerCP-conjugated streptavidin (BD, 554064) and antibodies against Sca-1, c-Kit, CD150 and CD48 as described under FACS protocol above. Labelled cells were fixed and permeabilized using Fixation/Permeabilization solution (BD, 554714) at 4°C for 20 min and washed with 1x BD Perm/Wash buffer (BD, 554714). Cells were next incubated in 1x BD Perm/Wash buffer containing AlexaFluor674-conjugated anti-Ki67 antibody (BioLegend, 652408, 16A8 clone; 1:100) or AlexaFluor674-conjugated anti-IgG2 antibody (BioLegend, 400526, RTK2758 clone; 1:100) at 4°C overnight. The next day, cells were washed with 1X BD Perm/Wash buffer and resuspend in 2% FBS containing 1xPBS. DAPI solution (Thermo, 62248; 1:500) was used to stain DNA before FACS analysis.
Instrument	Flow cytometry analysis was conducted on BD FACSCanto II Cell Analyzer (BD). Sorting was conducted on MoFlo Astrios Cell Sorter (BD) or MA900 Multi-Application Cell Sorter (Sony).
Software	Data was analyzed using FlowJo software.
Cell population abundance	Approximately 10-20x103 LSK+ and ~100x103 LSK- cells were sorted per mouse.
Gating strategy	For both flow cytometry analysis and sorting, cells were selected for live cell scatter in FSC/SSC then for singlets in FSCa/FSCh and for 7-AAD negativity (dead cell exclusion). Other markers were determined positive when signal was above unstained control. Isotype controls was used for Ki-67 staining.

- [x] Tick this box to confirm that a figure exemplifying the gating strategy is provided in the Supplementary Information.
